# Methane emissions in beef cattle: A review of measurements and mitigation strategies

**DOI:** 10.1016/j.vas.2026.100801

**Published:** 2026-08-04

**Authors:** Esther Taiwo, Gleise M. Silva, Ghader Manafiazar, Carolyn Fitzsimmons, Changxi Li

**Affiliations:** a Department of Agricultural, Food and Nutritional Science, University of Alberta, Edmonton, Alberta, Canada; b Department of Animal Science and Aquaculture, Faculty of Agriculture, Dalhousie University, Truro, Nova Scotia, Canada; c Lacombe Research and Development Centre, Agriculture and Agri-Food Canada, 6000 C&E Trail, Lacombe, Alberta, Canada

**Keywords:** Enteric methane emissions, Methane measurement methods, Mitigation strategies, Genetic selection and breeding, Beef cattle

## Abstract

Methane emissions from beef cattle make a considerable contribution to global greenhouse gas (GHG) emissions, leading to environmental, economic, and production challenges. This review presents the current understanding on enteric methane emissions in cattle, and evaluates the measurement techniques, bioindicators, and a range of management strategies to mitigate methane emissions, including dietary modifications, feed additives, vaccination, grazing strategies, and microbial manipulation, describing their respective benefits and limitations. The review also examines methane mitigation through genetic selection and breeding, including the applications of genome-wide association studies (GWAS) and genomic selection to identify and incorporate methane-reducing traits into the breeding programs. To facilitate genetic selection and breeding programs, more research is needed to link methane traits, gut microbes, and genetics to improve predictions of genetic merit of methane emissions on individual animals. While numerous mitigation strategies show promise, achieving lasting reductions in methane emissions requires an integrated approach that combines management practices with selective breeding and balances productivity, animal welfare, and both environmental and economic sustainability.

## Highlights

• Enteric fermentation in beef cattle is a source of greenhouse gases, with cow-calf producing up to 79% of enteric methane within the beef herd.
• Respiration chambers (gold standard), SF₆ tracers, GreenFeed**®**, and laser detectors are methods that can be used for direct measurement of methane emissions.
• Feed additives, dietary changes, and vaccines can reduce emissions by 7–98%, but the impacts cease once the treatment is discontinued.
• Methane traits are heritable (h^2^ = 0.10 – 0.45), enabling emission reduction through genetic selection and breeding.

## 1 Introduction

Greenhouse gas (GHG) emissions are increasing globally at a notable rate, leading to higher concentrations of carbon dioxide (CO_2_), methane (CH_4_), and nitrous oxide (N_2_O) in the atmosphere (World Meteorological Organization, 2024), thereby intensifying concerns about climate change and its severe implications for global food security. Agriculture contributes approximately 23% of global GHG emissions, with livestock production accounting for about 14.5% of the total global anthropogenic GHG emissions (Gerber et al., 2013; Crippa et al., 2021). Livestock emissions originate primarily from: a) CH₄ emissions from enteric fermentation in ruminants, b) CH₄ emissions from manure management, c) N₂O emissions from manure storage, fertilizer application, and feed production processes, and d) CO₂ emissions from other farm activities, such as fuel use in machinery and land management practices (Gerber & FAO, 2013; Grossi et al., 2019). Collectively, these emissions contribute between 5.6 and 7.5 Gt CO₂-eq annually and represent a major source of global agricultural emissions (Gerber et al., 2013; Herrero et al., 2016; Grossi et al., 2019)

Among livestock species, cattle are major contributors to GHG emissions, accounting for approximately 60% of livestock emissions in beef and dairy production systems and an estimated 61.7% of global livestock emissions (Bellarby et al., 2013; FAO, 2022). Enteric CH₄ generated during ruminal fermentation is the largest source of emissions in ruminant production systems (Legesse et al., 2016; Alemu et al., 2017). Although CH₄ remains in the atmosphere for only about 12 years, its global warming potential is 25–28 times greater than that of CO₂ over a 100-year period (IPCC, 2014). Consequently, enteric CH₄ remains a major target for climate change mitigation, although well-managed grazing systems may partially offset emissions through carbon sequestration (Gerber et al., 2013).

Beef cattle account for approximately 54.5% of global livestock CH₄ emissions, compared with 18.9% from dairy cattle (Food & Agriculture Organization of the United Nations [FAO], 2024). Within the beef cattle production system, enteric CH₄ is the leading source of GHG emissions, contributing about 63% of the total emissions, while CH₄ from manure contributes 13%, N_2_O from manure 19%, N_2_O from crop soil 1.39%, and CO_2_ from energy 3% (Chen et al., 2020b). In a comparison of enteric CH₄ emissions from different segments of the beef industry, the cow herd, bulls, calves, backgrounders, and finishers, Beauchemin et al. (2010) estimated that the cow-calf herd produced the highest level of enteric CH₄, accounting for 79% of the emissions. This was followed by finishers at 9%, backgrounders at 7%, bulls at 3%, and calves at 2%, highlighting that the cow herd is the primary contributor to CH₄ emissions in the beef production system. Enteric CH_4_ production in beef cattle also represents a significant loss of dietary energy, which is estimated at approximately 11%, thereby reducing overall production efficiency (Moraes et al., 2014). This inefficiency translates to economic losses for farmers and the beef industry.

Globally, enteric CH_4_ emissions from beef cattle increased by approximately 14% between 1990 and 2022, largely due to growth in cattle numbers and growing global demand for meat and dairy products (FAO, 2024). Nevertheless, improvements in cattle management and feed efficiency have shown the potential to reduce emissions intensity, as evidenced by the 17.5% reduction in the carbon footprint of Canadian beef production between 2013/2014 and 2021 (CRSB NBSA, 2024).

Significant progress has been made in CH₄ measurement technologies, management, and genomic selection strategies for reducing enteric CH₄ emissions in beef cattle. Consequently, an updated review is warranted to summarize current knowledge and identify emerging challenges and future directions for CH₄ mitigation. Therefore, this review provides an overview on measurement methods of CH₄ emissions, definitions of various CH₄ traits, and current management and genetic strategies for mitigating enteric CH₄ emissions in beef cattle.

## 2 Methane emission measurement

### 2.1 Direct measuring techniques

Direct CH₄ measurement techniques quantify CH₄ emissions by directly measuring exhaled or eructated CH₄, rather than estimating emissions from proxy or bioindicator traits. These methods mainly include the use of a respiratory chamber, sulfur hexafluoride (SF_6_), the laser CH_4_ detector method, GreenFeed**®** system, the sniffer method, and the portable accumulation chamber (PAC).

#### 2.1.1 Respiratory chamber (RC)

The RC offers a regulated setting for the collection and precise analysis of the animal's breath and gas production. This technology has been used for decades, and estimates CH_4_ emissions by measuring airflow through the chamber, and the difference in CH_4_ concentrations between incoming and outgoing air, generating approximately 4 –20 measurements per hour over 1–3 days (Pickering et al., 2015b). In most systems, a single gas analyzer alternates between chambers and sampling points at fixed intervals, resulting in periodic rather than continuous measurements (Garnsworthy et al., 2019). This method is considered the gold standard against which other techniques are compared because it continuously quantifies total enteric CH_4_ emissions by capturing all CH_4_ released through the mouth, nostrils, and hindgut, enabling accurate measurement of CH_4_ produced (Garnsworthy et al., 2019).

Despite its accuracy, RC has several practical limitations. It is expensive to install and operate, labor-intensive, and time-consuming, which limits the number of animals that can be measured and reduces its suitability for large-scale data collection . In addition, confinement within the chamber may alter normal feeding behavior, reduce feed intake, affect physiological responses, and influence productivity because the chamber environment differs from normal production conditions (Hegarty, 2004). Measurement variability among chambers can also occur due to differences in chamber design, airflow stability, calibration, and operating conditions, making comparisons across studies more difficult (Storm et al., 2012; Garnsworthy et al., 2019). Furthermore, respiration chambers have low throughput because animals are measured individually over several days, and they are generally unsuitable for grazing systems.

#### 2.1.2 Sulfur hexafluoride (SF₆) tracer gas method

The SF_6_ method was introduced as a method for measuring CH₄ emissions from individual animals under a less restrictive condition than RC, in the early 1990s (Johnson et al., 1994). This method can be used to estimate CH₄ emissions from cattle in both grazing and pen-based systems. The method involves placing a calibrated SF_6_ permeation tube into the rumen before the measurement period. The tube releases SF_6_ at a known rate, which allows CH₄ emissions to be estimated relative to the concentration of SF₆ recovered in breath samples. Sampling is usually conducted over 24 h and may be repeated for 5–10 days to improve reliability, or until the SF_6_ release rate from the permeation tube becomes less stable (McGinn et al., 2006). Muñoz et al. (2012) reported that SF_6_ permeation tubes may last for an average of 456 days, with an average release rate of 5.39 mg/day.

During sampling, a capillary tube is attached to the animal’s halter near the nostrils and connected to an evacuated canister, which collects breath samples from the mouth and nostril area over a 24-hour period. The capillary tube regulates airflow into the canister to prevent overfilling, with the canister typically reaching 50–70% capacity within 24 h (Garnsworthy et al., 2019). The collected gas samples are then analyzed using gas chromatography to determine CH₄ and SF_6_ concentrations. Methane emissions are calculated from the ratio of CH₄ to SF_6_ in the collected samples and the known SF_6_ release rate.

The SF_6_ tracer method is recommended over the RC method for large-scale studies involving numerous animals. It is less invasive and causes minimal discomfort to the animals. Muñoz et al. (2012) reported correlations of 0.69 for CH₄ production and 0.88 for CH₄ intensity between SF₆ and respiration chamber measurements, supporting its reliability. A limitation of this method includes possible overestimation when the SF_6_ permeation tube is nearly exhausted (Grainger et al., 2010). Measurements may also be affected when animals are close enough to contaminate each other’s breath samples, and daily canister replacement can disturb grazing behavior. In addition, the SF_6_ technique is labor-intensive and requires trained personnel and laboratory gas analysis.

#### 2.1.3 Laser methane detector

This is a portable device developed by Tokyo Gas Engineering Co. Ltd, Japan. It was first applied for CH_4_ measurement in 2009 by Chagunda et al. (2009). The laser CH_4_ detector is operated by directing the laser beam toward the animal’s nostril, with each measurement lasting approximately 15 s to 4 min (Chagunda et al., 2009; Ricci et al., 2014). The device estimates CH_4_ concentration in the air between the detector and the animal using infrared absorption spectroscopy, where CH_4_ molecules absorb infrared light emitted by a semiconductor laser. Changes in absorption around the CH_4_ spectral line are analyzed using wavelength modulation spectroscopy. Results are reported as CH₄ concentration parts per million-meter (ppm-m), volume percent meter (vol%m) or percent lower explosive limit per meter (%LEL·m) and can be exported electronically, using a tablet (Tokyo Gas Engineering Co., Ltd., 2013). The laser detector operates optimally within a temperature range of −17 °C to 50 °C and a relative humidity of 30% to 90% (Sorg et al., 2017). This method is non-invasive; it quickly detects CH₄ emissions (0.1 s) in various environments without direct contact with the animal, allowing measurements to be taken in the animal's normal environment. Chagunda et al. (2009) reported a correlation of 0.80 between CH_4_ emissions measured using the laser method and the RC method. The laser CH_4_ detector has some limitations; measurement accuracy can be affected by operator positioning, distance from the animal, animal head movement, nearby animals, and environmental conditions such as temperature (Sorg et al., 2017). In addition, because the system does not measure airflow, it provides CH_4_ concentrations rather than direct emission rates, which may limit its accuracy for estimating total CH₄ output. Its use may also be constrained by battery life, which typically lasts only 4 - 5 h (Sorg et al., 2017).

#### 2.1.4 GreenFeed® emission monitoring system (GEM)

The GreenFeed® emission monitoring system, developed by C-Lock Inc.®, was introduced around 2010 as an advanced technology for measuring CH₄ emissions in both indoor and outdoor production systems. It uses small amounts of pelleted feed to attract animals to the measurement station and encourage them to remain with their head inside the hood for approximately 3 - 7 min per visit (Hammond et al., 2016b; Garnsworthy et al., 2019). During this period, an extraction fan draws exhaled air through the hood, while airflow and gas concentrations are measured using infrared sensors. The system identifies each animal through its electronic tag, allowing CH_4_ emissions to be recorded at the individual-animal level. Measurements are typically collected over repeated visits, often 6 - 8 times within 24 h, and testing usually lasts 7 −14 days to obtain sufficient spot measurements, with a minimum of 20 visits, for reliable estimation of daily CH_4_ emissions (Manafiazar et al., 2016). The data collected are processed in real-time and made available through a web-based data management system, ensuring immediate and accurate monitoring of emissions. About 15 to 25 animals can be evaluated each day with this method using one GEM (Sorg et al., 2018).

Alemu et al. (2017) reported comparable CH₄ yield (g/kg dry matter intake [DMI]) between GEM and RC measurements, although daily CH_4_ production was slightly higher with GEM, likely due to differences in feeding conditions and reduced feed intake during RC measurements. The strong correlation between GEM and RC measurements (r = 0.92) reported by Huhtanen et al. (2019) also supports GEM as a reliable alternative for measuring CH_4_ emissions.

The GreenFeed**®** method is less invasive than RC and provides repeated breath measurements with real-time data processing. However, animals often require training to use the system, which may take up to two weeks, and some animals may not adapt to it (Hammond et al., 2016b). In addition, visit frequency can vary among animals due to differences in behavior, which may affect the number and consistency of CH_4_ records collected.

#### 2.1.5 Sniffer method

Another approach to measure enteric CH_4_ emission is the sniffer method (Garnsworthy et al., 2012a; Løvendahl et al., 2024), known for its cost-effectiveness and compatibility with milking systems. This method has been utilized in various studies and noted for its precision in quantifying CH₄ intensity (Difford et al., 2018a; van Engelen et al., 2018; Uemoto et al., 2024). The sniffer method involves installing CH₄ and CO₂ sensors, developed by companies such as Guardian NG and Edinburgh Instruments Ltd. (Livingston, UK), in automated milking machines or a feed (concentrate) dispenser. One end of a 3-meter-long polyethylene tube is connected to the enclosed feed bin of the machine to collect air samples while an animal consumes feed. The other end is attached to an infrared analyzer that measures CH₄ concentration, with each visit taking about 3 to 12 min. In total, approximately 40 to 80 animals can be evaluated each day by one feed dispenser (Sorg et al., 2018).

One of the advantages of this method is its capacity to evaluate a large number of animals quickly and frequently (Hammond et al., 2016b). It demonstrates a strong correlation of 0.79 with the respiratory chamber method for measuring CH_4_ emissions (Garnsworthy et al., 2012a). Different factors, such as the design of the feed bin and movements of the animal’s head during measurement, may influence the results. In addition, operational challenges, such as signal dilution caused by dust accumulation and sensor drift over time, may affect the performance of sniffers installed in feed bins, requiring routine maintenance and periodic calibration to ensure reliable measurements.

There have been recent developments in the application of sniffer-based CH₄ measurement technique, with several companies and research groups developing automated systems suitable for commercial livestock operations. For example, the UK-based company ZELP (Zero Emission Livestock Project) developed ZELP Sense, a wearable device that is designed to continuously measure CH_4_ and CO_2_ emissions from individual cattle, while also estimating DMI. The system is non-invasive, relatively inexpensive, provides real-time measurements, and has been successfully evaluated in cattle (Elmallah et al., 2024). Similarly, AgScent has developed the AgScent/Optiweigh unit, a sensor-based device that is integrated with an automated livestock weighing technology to measure CH₄ and CO₂ from the exhaled breath of individual animals (AgScent Pty Ltd., 2026; Senevirathne et al., 2025). Another example is the Moologger system (Tecnosens, Italy), which measures CH_4_ and CO₂ concentrations in exhaled breath and can be integrated into automated milking or feeding systems for continuous data collection (Stratz et al., 2025). Although still under evaluation, these technologies are a cost-effective alternative to traditional CH₄ measurement methods and are suitable for large-scale implementation, supporting phenotyping, genetic evaluation, and assessment of mitigation strategies in commercial production systems.

#### 2.1.6 Portable accumulation chambers (PAC)

A portable accumulation chamber can be used to measure CH₄ emissions in small ruminants, and more recently in cattle (Bilton et al., 2025; Goopy et al., 2011). In this method, an animal is confined within an enclosed chamber for a short period (about 40– 60 min), during which respired and eructated gases accumulate, and are subsequently measured to estimate CH_4_ emissions (Jonker et al., 2020; Robinson et al., 2015).

One advantage of the PAC method is that it can be used for large-scale CH_4_ measurements, with the capacity to evaluate approximately 84 animals per day (Jonker et al., 2020). In addition, PAC measurements have demonstrated moderate repeatability estimates ranging from 0.33 to 0.55 and moderate to strong genetic correlations (0.62–0.67) with RC measurements, supporting their use as a reliable and cost-effective method for CH_4_ measurement (Jonker et al., 2018). However, because measurements are collected over a short period, PAC estimates may be influenced by the timing of feeding, or animal activity, increasing measurement variability. Therefore, a standardized measurement protocol should be strictly followed, and appropriate statistical standardization should be applied during data analysis to account for variation associated with differences in time off feed and other technical factors.

#### 2.1.7 In vitro techniques

The in vitro technique for measuring CH_4_ emissions provides a laboratory-based method for assessing CH_4_ production under controlled conditions. This method serves as an alternative to direct CH_4_ measurement and can be used as an initial screening tool to assess the effectiveness of various dietary compounds, such as essential oils, metabolites, and tannins, in reducing methanogenesis (Tedeschi et al., 2021). The technique involves mixing the feed materials with ruminal fluid from an animal and a buffer solution, then incubating the mixture at 39 °C. This environment allows rumen microbes to ferment the feed over a period ranging from 24 to 144 h, enabling researchers to observe and measure CH_4_ production under controlled conditions (Storm et al., 2012). The amount of gas produced is measured and the composition is analyzed to determine CH_4_ production. There are different in vitro techniques used for CH_4_ measurement, for example, syringes (Blümmel & Ørskov, 1993), the rusitec method (Czerkawski & Breckenridge, 1977), the dual-flow continuous culture system (Hoover & Stokes, 1991), the batch culture system (Mauricio et al., 1999), and the fully automated system (Pellikaan et al., 2011). Some factors that may influence the result of this technique include rumen fluid donor characteristics, the feedstuff incubated, the buffer solution and incubation process, and the collection and processing of inoculant (Yáñez-Ruiz et al., 2016).

Danielsson et al. (2017) found a strong correlation (r = 0.98) between the in vitro CH₄ measurement and the GEM. One advantage of this method is that a large number of samples can be screened within a relatively short period of time (1- 4 weeks; Storm et al., 2012), and the lower cost compared to in vivo techniques, allowing for replication. One limitation of this technique is that rumen microbes in the collected fluid may not be adapted to the test feed if the donor animal was not previously exposed to that diet. This can affect fermentation patterns and CH₄ estimates. This issue can be minimized if donor animals are adapted to the feed prior to rumen fluid collection. Also, the technique only replicates the process of rumen fermentation (Storm et al., 2012), and it does not account for the whole digestion process that may influence CH_4_ production, such as the passage rate, absorption, and interaction with other food items, with the results differing from in vivo measurements.

## 3 Estimating methane emissions from correlated traits and bioindicators

Direct measurement of methane emissions is constrained by the high cost and limited capacity of current measurement methods, restricting their application for large-scale phenotyping. As a result, correlated traits and other indirect approaches are increasingly used to estimate CH_4_ emissions in larger animal populations. However, according to Storm et al. (2012), measuring CH_4_ emission directly from animals may achieve error below 5 - 10%, while techniques that estimate CH_4_ emissions based on correlated traits have prediction errors that range between 10 and 25%.

### 3.1 Predicted methane emission (PME) from correlated traits

Predicted methane emissions can be estimated from certain correlated traits such as feed intake, body weight, total digestible nutrients, and milk production. Predicted CH₄ emission does not require any machine or equipment to estimate emissions, other than using information from traits that could be measured on the farm (Kebreab et al., 2015), making it an easier method to use. However, one disadvantage of this PME method is that the prediction equation relies on information from traits that might not be readily available, such as feed intake. Predicted CH₄ emission models operate under the assumption that CH₄ emission per unit of feed gross energy remains constant, regardless of an animal's potential to ferment feed, and do not offer individualized information beyond variations in feed intake (de Haas et al., 2011; Lakamp et al., 2022). This assumption has major limitations. Animals with the same amount of feed intake, for example, may have diverse feeding patterns or appetites, which may result in different CH₄ emissions. Failing to consider the variations can significantly impact the prediction precision of CH_4_ emissions and potentially lead to inconsistencies in estimated results.

### 3.2 Potential bioindicators to estimate methane emissions

#### 3.2.1 Rumen microbiome

The rumen microbiota plays a crucial role in CH₄ production in cattle. H_2_ and CO_2_ that are primarily generated by rumen microbes through fermentation are utilized by methanogenic archaea to produce CH₄ (Wallace et al., 2017). Certain rumen archaeal species, such as those from the *Methanomassilicoccales* order, have been reported to enhance CH₄ production, especially in the presence of hydrogen-producing microbes (Borrel et al., 2014; Difford et al., 2018a). Similarly, Difford et al. (2018a) propose that the genus *Sporobacter* found in their study may also contribute to CH₄ production following the same course. Moreover, some bacterial species, including *Sphaerochaeta* and members of the BS11 Group, have also been positively linked to increased CH₄ production (Difford et al., 2018a), as their fermentation processes produce CO_2_ and H_2_ (Caro-Quintero et al., 2012; Wang et al., 2016). This implies that animals harboring these microbes are likely to produce increased CH₄ emissions.

Roehe et al. (2016) identified twenty microbial genes associated with CH₄ yield in beef cattle, demonstrating the genetic basis of methanogenesis at the microbial level. Their study also revealed a genetic component in the rumen microbial population using 16S rRNA sequencing, with evidence of sire effect on the microbial community composition. Difford et al. (2018a) detected specific bacterial and archaeal species associated with CH₄ production that showed heritable traits, using 16S rRNA, which indicates that the host genetics plays a significant role in shaping the composition of the rumen microbiota. Employing whole metagenome analysis using nanopore technology, Wallace et al. (2019) uncovered a diverse array of archaea, anaerobic bacteria, fungi, and protists in the rumen ecosystem. This comprehensive approach provides a more detailed picture of the microbial players involved in methanogenesis. Difford et al. (2018a) reported that CH₄ emission is influenced by a complex interplay of factors, including the composition of the rumen microbiome, the host animal's genetics, and the interactions between these elements. Due to the correlation of rumen microbiome with CH4 emissions, CH₄ yield was predicted based on the abundance of rumen microbiota from animals fed different diets (fresh cut grass and high concentrate diet), with a reasonable prediction accuracy of 0.66 and 0.85, respectively, in beef cattle (Miller et al., 2023). A study by Zhang et al. (2023) found that including rumen microbiota as a predictor in the CH₄ prediction model improved accuracy, and this was revealed by a decrease in the mean absolute error and the root mean square error by up to 13.31% and 11.12%, respectively, for CH₄ yield (g/kg DMI), and 5.08% and 3.80% respectively, for CH₄ production (g/d).

#### 3.2.2 Fecal methanogen

Research has identified promising alternative methods for predicting CH_4_ emissions in cattle using fecal samples. Gill et al. (2011) and McCartney et al. (2013b) demonstrated that archaeol concentration in fecal matter can serve as a reliable proxy for CH₄ production. Archaeol, a membrane lipid specific to archaea, can be quantified using gas chromatography-mass spectrometry (GC–MS) to estimate methanogen abundance in the rumen (McCartney et al., 2013a). Gill et al. (2011) reported a positive correlation (r = 0.37) between CH_4_ production and archaeol concentration in fecal samples. In a deeper analysis, Manafiazar et al. (2021) demonstrated that fecal microbial profiles can be used to predict daily CH₄ emission with an *R*^2^ of 0.53. The study used amplicon sequencing method of the fecal microbiota to identify microbial populations associated with CH_4_ emissions (production). They found that higher relative abundance of *Methanobrevibacter wolinii* and lower *Methanosphaera* species ISO3-F5 abundance to be associated with low CH₄ emission. This relationship provides a valuable tool for researchers and potentially for farmers to estimate CH_4_ emissions.

#### 3.2.3 Saliva and oral fluid /Swabs

Saliva and oral fluid sampling are increasingly being investigated as a non-invasive tool to study oral and rumen-linked microbial communities (Adams et al., 2025; Young et al., 2020; Tapio et al., 2016). Samples may contain rumen microbes introduced during rumination and regurgitation and therefore, partially reflect rumen microbial composition, making them useful for estimating CH₄ emissions (Ong et al., 2026). The samples are collected using oral swabs or saliva collection devices, and microbiome DNA is then extracted. Adams et al. (2025) and Young et al. (2020) supported the use of bovine oral and buccal sampling as a proxy for rumen microbial composition, although differences between oral and rumen microbial populations were still observed. More recently, Ong et al. (2026) added that oral microbiome profiles may be useful for predicting CH₄ emissions in grazing animals.

Compared with rumen fluid collection methods such as cannulation or stomach tubing, saliva sampling is simpler, faster, and less stressful for animals, making it more practical under commercial production conditions. Despite these advantages, several limitations remain. Oral microbial communities may differ substantially from rumen microbial populations, and saliva composition may be influenced by factors such as diet, feeding behavior, sampling time, and environmental exposure (Adams et al., 2025; Young et al., 2020).

#### 3.2.4 Milk composition

Milk composition has emerged as a promising proxy for estimating CH_4_ emissions in cattle. Research suggests that the fatty acid content in milk samples correlates with the abundance of methanogenic archaea in the rumen, as well as with feed composition (Dijkstra et al., 2011; Montoya et al., 2011; Dehareng et al., 2012). This relationship provides a foundation for using milk composition as a potential bioindicator of CH_4_ production. The fat content in milk has been identified as a particularly useful marker (Moraes et al., 2014), and this approach shows promise not only in dairy cattle, where milk sampling is a routine practice, but also in beef cattle where milk samples can be collected from lactating cows during their nursing period. The application to beef cattle is noteworthy, as it is a non-invasive method for estimating CH₄ emissions in a sector where direct measurements can be more challenging.

A study by McParland et al. (2024) predicted CH_4_ emission using mid-infrared (MIR) spectral analysis, a method that assesses the absorption of mid-infrared light by a sample to reveal its chemical composition, which can be used for CH₄ prediction. They recorded a correlation range of 0.55 to 0.71 between the predicted CH₄ production and the actual CH₄ production. Bittante and Bergamaschi (2019) predicted CH_4_ yield (g/kg DMI) with a range of 16.77 to 17.11 and CH_4_ intensity (g/kg Corrected Milk) of 11.21 to 11.66 using fatty acids profiles from four different milk sample types, including morning whole milk, evening whole milk, partially skimmed evening milk, and vat milk. These CH_4_ estimates are highly correlated (r > 0.8) with those obtained from the reference bovine milk sample collected from both the morning and evening milkings, which served as the standard against which other samples are evaluated. The findings infer that milk composition could be an efficient proxy for estimating CH_4_ emissions, with a reasonable level of prediction accuracy.

### 3.3 Spectroscopic methods for methane prediction

Some indirect methods of CH_4_ measurement include Fecal Near-infrared Reflectance Spectroscopy (FNIRS) and Fourier Transform Infrared (FTIR). Near-infrared reflectance spectroscopy (NIRS) is a method used to quantify the composition of feces or feed using near-infrared light, which measures how specific wavelengths are reflected. According to González et al. (2014) fecal samples are collected from animals at multiple time points to ensure a comprehensive representation of dietary variations. The samples are preserved in ice and then frozen to maintain the integrity of the sample pending analysis. The samples are then thawed and dried at 60 °C for 48 h, ground to a fine consistency to ensure homogeneity and optimal conditions for the NIRS scanning. The sample can then be analyzed using a NIRS spectrometer, and the resulting spectra are processed with calibration equations to estimate the important nutritional parameters such as dry matter digestibility, nitrogen content and the proportion of the non-grass species in the diet (Coates & Dixon, 2011). The dry matter digestibility obtained from FNIRS, and the feed intake are then used to predict CH_4_ emissions using a method from the Commonwealth of Australia (2014).

Fourier Transform Infrared (FTIR) spectroscopy examines the chemical profile of milk, revealing variation in fatq; protein, and metabolite levels (van Gastelen et al., 2018). These variations reflect differences in how cows digest their feed and produce CH_4_, and by examining this milk component, a cow’s CH_4_ emissions can be estimated (Dehareng et al., 2012; Vanlierde et al., 2015). Fourier Transform Infrared (FTIR) spectroscopy makes use of mid-infrared light to scan a sample, the infrared beam passes through or reflects off the sample, and certain wavelengths of light are absorbed by the molecules (Villar-Hernández et al., 2023). The strength of absorption at specific wavenumbers reveals the sample’s chemical makeup. This absorption pattern serves to indicate the presence of particular compounds such as milk metabolites that can be linked to the amount of CH₄ a cow produces.

Vanlierde et al. (2018) found coefficients of determination of 0.65 (calibration) and 0.57 (cross-validation) when comparing milk FTIR predictions with chamber-based CH₄ measurements. Similarly, Vanlierde et al. (2021) reported that a predictive model that used FTIR had a coefficient of determination of 0.68 in cross-validation, with a standard error of 57 g CH₄/day when a combined dataset from SF₆ tracer and RC data was used as the reference.

Accurate estimation of absolute CH₄ production is important for evaluating nutritional and management-based mitigation strategies, carbon accounting, and environmental assessments. Measurement methods such as respiration chambers and the SF₆ tracer technique are therefore commonly regarded as more suitable for estimating absolute CH₄ emissions because they provide relatively accurate quantification of total CH4 output over time (Hammond et al., 2016a; Hristov et al., 2018). GreenFeed® systems may also provide reasonably accurate CH₄ estimates when repeated measurements are obtained under controlled conditions (Huhtanen et al., 2015, 2019). In contrast, breeding programs primarily require methods that can reliably classify and rank animals according to their CH₄ emissions rather than precisely quantify absolute CH₄ output. Given the large number of animals involved in CH₄ phenotyping, short-term measurement approaches and indirect proxy methods, including sniffer systems, laser CH₄ detectors, PAC, milk composition traits, fecal biomarkers, microbiome profiles, are often more practical for genetic evaluation (De Haas et al., 2011; Negussie et al., 2017; Difford et al., 2018b; Wahinya et al., 2022; Ong et al., 2026). Table 1 summarizes the main CH₄ measurement methods and prediction approaches described in this review and compares their suitability for estimating absolute CH₄ emissions and ranking animals for genetic evaluation, accuracy, commercial suitability, and cost.

**Table 1:** Comparison of direct and prediction methods for estimating methane emissions.

**Method**	**Measurement type**	**Primary application**	**Accuracy**	**Commercial suitability**	**Invasiveness**	**Relative cost**
Respiration chamber	Direct	AM and AR	Very high	Low	High	Very high
SF₆ tracer technique	Direct	AM and AR	High - Very high	Limited	Moderate	High
GreenFeed**®**	Direct	AM and AR	High	Limited	Low	High
Sniffer systems	Direct	AR	Moderate	Potentially	Low	Moderate
Laser methane detector	Direct	AR	Moderate	Potentially	Low	Moderate
Portable accumulation chamber	Direct	AR	Moderate	Potentially	Low - Moderate	Moderate
Milk fatty acids	Indirect	AR	Moderate	Potentially	None	Low
Fecal biomarkers	Indirect	AR	Moderate	Potentially	Low	Low
Rumen microbiome	Indirect	AR	Moderate	Limited	Moderate	Moderate
Oral microbiome	Indirect	AR	Moderate	Potentially	Low	Moderate

AM = Absolute Methane Estimation, AR = Animal Ranking. Commercial suitability was evaluated based on current commercial adoption, cost, infrastructure requirements, and practicality for implementation in livestock production. The ratings are based on the authors' assessment of the current literature and may change as technologies evolve.

As more research related to CH_4_ emissions in cattle is conducted, additional novel and reliable measurement methods are expected to emerge. The choice of measurement method influences not only the precision of emission estimates but also the traits that can be derived for management and breeding applications. Consequently, understanding CH_4_ traits is essential for interpreting the phenotype records and developing effective mitigation strategies.

## 4 Reporting methane emissions

Methane emissions are being reported in several ways, each offering distinct perspectives on the animal's CH_4_ emissions profile.

### 4.1 Methane production

Methane production is defined as the amount of CH_4_ emitted per unit of time, usually expressed in grams per day (g/d) or liters per day (L/d). Methane production values vary depending on several factors such as DMI, diet type, animal type/genetics (beef or dairy), physiological stage, and measurement techniques (Hegarty, 2007; Donoghue et al., 2013; Hayes et al., 2016). Using RC, Donoghue et al. (2013) reported CH₄ production ranging from 122 to 350 L/day in young Australian Angus bulls and heifers, while Hayes et al. (2016) reported values ranging from 78.9 to 250.9 L/day (mean = 132.6 ± 25.5 L/day) in Angus population used for genomic evaluation. In contrast, Hegarty et al. (2007), using the SF₆ tracer technique, reported CH₄ production ranging from 142.3 to 190.2 g/day in Angus steers. However, a direct comparison of measurement methods by Kamalanathan et al. (2023) showed that RC (459 ± 6.5 g/day) and GreenFeed® (433 ± 8.7 g/day) produced comparable mean CH₄ production estimates in Holstein dairy cattle.

Reporting CH_4_ emissions in g/day or L/d is a simple and direct measure of the total CH_4_ an animal emits per day and can be useful as a baseline metric for reporting CH_4_ emissions. However, CH_4_ production (g/day or L/d) is highly correlated with DMI and body weight (Renand et al., 2019; Donoghue et al., 2016; Manzanilla-Pech et al., 2016), and therefore may not provide a comprehensive understanding of emission efficiency. Consequently, selecting animals based solely on lower CH_4_ emissions per day could unintentionally favor animals with lower feed intake and productivity, which may not support optimal production and environmental performance.

### 4.2 Methane yield

Methane yield is defined as the amount of CH_4_ emitted per unit of feed consumed or per kilogram (kg) of dry matter intake. Methane yield may be influenced by animal individual variability, diet composition, feed intake levels, or management practices. Cattle fed restricted forage-based diets consisting primarily of alfalfa and oaten hay chaff produced CH₄ yields ranging from 13.1 to 29.5 g/kg DMI (Arthur et al., 2018; Hayes et al., 2016). In contrast, Holstein dairy cows offered ad libitum total mixed rations produced CH₄ yields ranging from 5.1 to 75.7 g/kg DMI. Similarly, dairy cows fed diets based on processed barley, rapeseed meal, corn silage, soybean meal, and grass-clover silage exhibited CH₄ yields ranging from 3.7 to 35.8 g/kg DMI (Manzanilla-Pech et al., 2022). An advantage of using the CH_4_ yield is that it accounts for feed intake, making it useful for comparing animals across production systems or different feed intake levels. However, CH_4_ yield does not account for differences in productivity, such as body weight or milk production. Therefore, CH_4_ yield should be interpreted with CH_4_ production to accurately assess an animal’s environmental impact, as an animal with a low CH_4_ yield but high feed intake may produce more CH_4_ than an animal with a higher CH_4_ yield but lower feed intake.

### 4.3 Methane intensity

Methane intensity refers to the amount of enteric CH_4_ released per unit of animal product, typically expressed as grams of CH_4_ per kilogram of product, such as body weight, beef produced, or per liter of milk. In young beef cattle, CH_4_ intensity ranged from 25.8 to 67.8 L/kg body weight with a standard deviation (SD) of 9.4 (Donoghue et al., 2013). In Holstein dairy cows, CH₄ intensity ranged from 4.1 to 29.7 g/kg milk, with SD ranging from 3.6 to 3.9 (Kamalanathan et al., 2023), while values ranging from 1.9 to 29.5 g/kg milk with a SD of 2.3 were reported by Manzanilla-Pech et al. (2022). Recent studies (Kandel et al., 2018; Flay et al., 2019; González-Recio et al., 2020) have shown that focusing solely on production traits in breeding programs can increase overall CH_4_ production while decreasing CH_4_ intensity, due to improved productivity. However, explicitly including CH_4_ intensity in selection indices results in more efficient animals that emit less CH_4_ per unit of product. This measure can be used when the aim is to enhance the environmental sustainability of production systems by lowering CH_4_ emissions per unit of milk or animal product rather than the total emissions from the animals.

### 4.4 Residual methane production

Residual methane emission is calculated as observed CH_4_ emission minus the predicted CH_4_ emission. It is a good method to quantify CH₄ and has been proposed as a valuable trait for selection (Herd et al., 2014). Predicted CH_4_ emission can be obtained using a multiple linear regression model that considers various factors, such as DMI, and body weight, which can influence CH_4_ emissions. Residual methane production is then calculated as the residuals from this model, representing the difference between the observed CH_4_ emission and the emissions predicted by the model based on these parameters (Smith et al., 2021). Smith et al. (2021) found a significant difference in CH_4_ production between beef cattle with low and high residual CH_4_ emissions. Their research revealed that cattle classified as low emitters produced 30.4% less CH_4_ daily compared to their high emitters, while the DMI and average daily gain did not differ significantly. Furthermore, the study identified strong correlations between residual CH₄ emission with other calculated CH₄ metrics, including daily CH_4_ emission, CH_4_ intensity, and CH_4_ yield with r = 0.86, 0.86, and 0.89, respectively. It is suggested to be a better selection trait option for CH_4_ emissions in cattle than CH_4_ yield and production because, according to Donoghue et al. (2016) and Smith et al. (2021), residual CH_4_ production does not have adverse correlations with production traits.

## 5 Methane mitigation strategies

Reducing enteric CH₄ emissions is important for improving the environmental sustainability of beef production, and CH₄ can be mitigated through management practices and selective breeding. Dietary modification and improving feed efficiency are CH₄ mitigation strategies that have been researched previously (Martin et al., 2010; Beauchemin et al., 2008; Machmüller et al., 2000). Subsequent research used feed additives, microbial interventions, vaccination, and grazing management, and more recent studies have shifted toward host genetic selection and microbiome-targeted mitigation (Garcia-Ascolani et al., 2024; Muntari et al., 2023; Asselstine et al., 2021; Melgar et al., 2020; Wallace et al., 2019).

### 5.1 Mitigating methane emissions through management

Over the past decades, mitigating CH_4_ emission through management practices has become an important focus for researchers, leading to notable advancements in the development and adoption of practical mitigation strategies such as the use of feed additives, vaccines, diet modification, grazing systems, and manipulating rumen microbes (Wright, 2004; Beauchemin et al., 2008; Janssen, 2010; Veneman et al., 2016; Savian et al., 2018). Although the effectiveness of these approaches varies, they have shown promising results, usually ranging from about 10% to a 70% reduction in CH_4_ emissions (Appuhamy et al., 2013;ERA, 2024).

#### 5.1.1 Diet modification

The composition of the diet significantly affects the rumen environment and the CH_4_-producing archaea (Martin et al., 2010), which are playing a crucial role in CH_4_ emissions. Cattle primarily consume forages such as grasses and legumes, which are rich in complex carbohydrates like cellulose and hemicellulose. These structural carbohydrates require extensive microbial fermentation in the rumen, a process that generates CH₄ as a by-product (Janssen, 2010). In contrast, diets low in fiber and high in simple sugars, such as high-grain rations or young, highly fermentable forages, are more digestible and shift ruminal fermentation patterns by increasing both the total production and relative proportions of volatile fatty acids. This shift favors propionate synthesis, an H₂-utilizing pathway, thereby decreasing CH₄ emissions (Hills et al., 2015; Chen et al., 2020a). Higher propionate production lowers the availability of H₂ for methanogenesis, which further suppresses CH₄ formation (Beauchemin et al., 2009). Although high-grain diets initially increase H₂ production and partial pressure in the rumen, the subsequent stimulation of propionate pathways ultimately reduces overall H₂ formation and limits CH₄ synthesis (Janssen, 2010). Similarly, starch-rich diets, such as those based on cereal forages, reduce acetate production and promote propionate formation, contributing to lower CH₄ emissions (Beauchemin et al., 2008).

In addition to carbohydrate manipulation, dietary lipids can also mitigate CH₄ production by 10% to 39% (Machmüller et al., 2000; Jordan et al., 2006; Beauchemin et al., 2009). Diets high in oil reduce CH₄ emissions because unsaturated fatty acids inhibit methanogenic archaea and act as alternative H₂ sinks through biohydrogenation (Machmüller et al., 2003; Chaves et al., 2008). Although low-fiber, high-grain diets can reduce CH₄ production in cattle, they may not be economically feasible across all phases of beef production. Furthermore, supplementing grazing animals with high-oil diets can be challenging, as elevated lipid levels may negatively affect fiber digestibility (Machmüller et al., 2000).

#### 5.1.2 Feed additives

Feed additives are substances added to animal diets to enhance feed digestion and utilization quality, boost animal performance, and promote overall animal health (European Food Safety Authority, 2026). Feed additives used as CH_4_ emissions mitigators can be classified into CH_4_ inhibitors and rumen fermentation modifiers. Methane inhibitors, such as three-nitrooxypropanol (3-NOP), and *Asparagopsis taxiformis* indirectly prevent methanogenesis, while rumen fermentation modifiers, like ionophores, essential oils, and tannins, alter the rumen environment to make it less favorable for CH_4_ production by obstructing the availability of H_2_ and other reactants needed for methanogenesis, or by reducing the methanogenic microbiota (Almeida et al., 2021). Feed additives have been identified as one of the most effective strategies for mitigating CH_4_, with reported reductions of up to 80% in CH_4_ emissions (Roque et al., 2021).

Supplementing beef cattle diets with varying levels of *Asparagopsis taxiformis*, a type of macroalgae, resulted in up to a 98% reduction in CH_4_ yield, without compromising production (Kinley et al., 2020). Likewise, Roque et al. (2021) reported a 45% and 68% reduction in CH_4_ yield by supplementing beef steer diets with 0.25% and 0.50% *Asparagopsis taxiformis*, respectively. However, the use of algae raises concerns due to the toxic nature of the bromoform; a naturally occurring halogenated compound that is produced by *Asparagopsis taxiformis,* but some authors reported low negative effects of bromoform on the animals and the environment (Kinley et al., 2020; Muizelaar et al., 2021; Roque et al., 2021). There is also a concern about the accumulation of heavy metals and high iodine levels in algae, which may cause health risks to animals and raise food safety concerns when consumed in large quantities (Makkar et al., 2016; Kim et al., 2019).

Similarly, supplementation of beef diets with ionophores, such as monensin and lasalocid, led to a 30% and 27.5% reduction in CH_4_ emissions in cattle fed high and low-concentrate diets, respectively (Guan et al., 2006). Similarly, Appuhamy et al. (2013) reported a 10.7% reduction in CH_4_ production in beef steers using monensin. However, the effectiveness of ionophores may reduce over time due to the development of antimicrobial resistance (Beauchemin et al., 2022).

The use of 3-nitrooxypropanol (3-NOP) has consistently led to reduced enteric CH₄ emissions without adversely affecting animal performance. For example, Melgar et al. (2020) reported a 27% reduction in CH_4_ production in dairy cattle supplemented with 3-NOP. The effectiveness of 3-NOP is influenced by diet composition, especially neutral detergent fiber (NDF) concentration. Dairy cattle typically consume lower-NDF diets, which enhances the antimethanogenic effect of 3-NOP, whereas the higher-NDF diets commonly fed to beef cattle may reduce its efficacy (Dijkstra et al., 2018). Nevertheless, Alemu et al. (2021) reported a 76% reduction in CH_4_ production when 3-NOP was included at 107.6 mg/kg DM in a corn-based finishing diet (increased NDF), demonstrating its potential as an effective mitigation strategy in commercial beef feedlots.

One of the limitations of 3-NOP is that animals fed diets supplemented with 3-NOP have been reported to release about 10-fold higher hydrogen compared to those fed diets that do not contain 3-NOP, and this could have an implication on their total gas emission (Guyader et al., 2017; Melgar et al., 2020). In addition, reductions in dry matter intake have been reported in some beef cattle studies (Kim et al., 2020a; Alemu et al., 2021), which could affect animal performance. Furthermore, the antimethanogenic effect of 3-NOP is not permanent and diminishes once supplementation ceases, requiring continuous dietary inclusion to maintain CH_4_ reductions (Hristov et al., 2015).

#### 5.1.3 Manipulation of rumen microbial communities

The community of microbes in the rumen (archaea, protozoa, bacteria, mycoplasmas, anaerobic organisms, and viruses) is influenced by various factors, including the animal's genetics, age, health status, season, feed composition, location, and the use of antibiotics (Henderson et al., 2015; Tapio et al., 2017).

Altering the rumen microbial components can help mitigate CH_4_ production (Janssen, 2010). This can be achieved by using live microbial products fed directly to the animals to influence the microbial population. These live microbial products are referred to as Direct-Fed Microbials (Krehbiel et al., 2003), which includes propionic acid bacteria (propionibacteris), acetogens (homoacetogens), or CH_4_ oxidizing bacteria. The propionic acid bacteria promote the production of propionate, using the H_2_ available in the rumen to make it less available for the production of CH_4_. Examples include *Propionibacterium acidipropionici, Propionibacterium japonicas, and Propionibacterium freudenreichii* (Vyas et al., 2016). The study by Chen et al. (2020a) found that propionic acid bacteria increase the production of propionate, resulting in a 20% reduction in CH_4_ emissions in dairy cattle. Vyas et al. (2016), however, did not find a reduction in CH_4_ emissions using propionic acid bacteria. Acetogens also help to reduce CO_2_ and H_2_ in the rumen to produce acetate (Jeyanathan et al., 2014). In addition, Kim et al. (2020b) reported that certain isolated homoacetogen strains such as *Proteiniphilum acetati-genes, P. acetatigenes and Alkaliphilus crotonatoxidans* have the ability to reduce CH_4_ production in vitro.

Defaunation is a process aimed at eliminating protozoa from the rumen. This technique, implemented through chemical treatments or dietary modifications, has been shown to reduce CH_4_ production by up to 11% (Newbold et al., 2015). Protozoa in the rumen produce H_2_ as a by-product during the fermentation of feed particles, which the methanogenic archaea utilize for CH_4_ production (Toyber et al., 2024). Removing protozoa reduces the overall H₂ production, thereby limiting the H₂ availability for methanogenic archaea to produce CH_4_ in the rumen. While effective, the long-term impacts of defaunation on animal health and productivity require further research.

A study by Garcia-Ascolani et al. (2024) investigated the use of avian polyclonal antibodies targeting *Methanobrevibacter gottschalkii* Ho and *M. ruminantium* M1, aiming to examine the effect on ruminal fermentation and CH_4_ production. The research found that supplementing with these antibodies reduced CH_4_ production ex vivo and led to increased propionate production, consequently reducing CH_4_ production. A challenge of this method is that, once the rumen microbiome is fully established in mature animals, it becomes resilient, maintaining its structure and function, making it difficult to manipulate (Yáñez-Ruiz et al., 2015).

#### 5.1.4 Use of vaccination

The use of vaccination to reduce CH_4_ emissions has been investigated as a promising strategy for mitigating enteric CH_4_ production in cattle. Vaccines can be used to stimulate the immune system to produce antibodies in the saliva. The antibodies migrate into the rumen, where they inhibit or neutralize methanogen activity and thereby reduce CH₄ emissions in cattle (Subharat et al., 2015). Wright (2004) investigated the impact of vaccination on reducing methanogenesis in the rumen of sheep, reporting a 7.7% reduction in CH₄ emissions. This reduction was achieved in sheep treated with two doses of a vaccine administered 153 days apart, which included three methanogen strains from *Methanobrevibacter* species (ZA-10, 1Y, and AK-87). Williams et al. (2009) also observed a reduction in the mean density of methanogens although the decrease was not statistically significant. They also reported an increase in the level of methanogen-specific IgG, and a change in the methanogen population, with the use of 3 doses (days 0, 28, and 103) of vaccine prepared from five methanogens (*Methanobrevibacter* ZA-10^T^, AK-87, 1Y, *Methanosphaera stadtmanae* MCB-3^T^, and *Methanomicrobium mobile* BP^T^). Their result suggested that some methanogen species were susceptible to the vaccine.

Doubts have been raised about the success of the use of vaccination, due to uncertainty regarding the survival of antibodies in the rumen. However, studies have demonstrated that antibodies can remain stable in the rumen (Williams et al., 2008; Subharat et al., 2015). Subharat et al. (2015) conducted a study where cattle were vaccinated with a recombinant glycosyl transferase protein derived from *Methanobrevibacter ruminantium*. The vaccinated cattle produced antigen-specific antibodies in their saliva, which were then passed into the rumen and remained stable for about 4 to 8 h. As only a few studies (Muntari et al., 2023, 2024) have been conducted on cattle, there is a need for more research to confirm the effectiveness of vaccination in mitigating CH₄ emissions in cattle. Also, it is crucial to identify the appropriate adjuvant to stimulate a strong antibody response.

#### 5.1.5 Grazing management

Grazing management has emerged as a promising strategy for mitigating CH₄ emissions in ruminant livestock systems (DeRamus et al., 2003; Savian et al., 2014, 2018). Proper grazing management can maintain pastures in a vegetative state, improving digestibility and potentially reducing CH₄ emissions per unit of feed intake (Beauchemin et al., 2008).

Two primary grazing methods, continuous and rotational grazing, have different implications for CH₄ mitigation. Continuous grazing allows unrestricted access to a pasture area for the entire grazing period, enabling animals to selectively feed on the most nutritious plants. While this enhances grazing preference, it can lead to overgrazing of preferred species and underutilization of less palatable plants (Savian et al., 2014). Over time, this method may result in suboptimal forage quality, potentially leading to higher CH₄ emissions per unit of animal product.

Rotational grazing, in contrast, involves dividing pastures into smaller paddocks and systematically moving animals between them. This method ensures more efficient utilization of all forages in each paddock (Hancock, 2009), and may lead to reduced CH₄ emissions (Hristov et al., 2013). Rotational grazing allows time for plant regrowth before being grazed again, maintaining higher forage nutritive value (Norton, 2022). Better forage quality and availability can enhance animal performance, potentially reducing the time to market for beef cattle or increasing milk production in dairy cows, thus lowering CH₄ emissions intensity (Hristov et al., 2013).

Research findings on the effectiveness of these grazing methods in reducing CH_4_ emissions have been mixed. Savian et al. (2014) reported that continuous grazing reduced CH₄ intensity compared to rotational grazing. The lower CH₄ intensity likely resulted from greater daily DMI and improved productivity under continuous stocking, where grazing pressure is more evenly spread, and animals can choose better-quality forage. However, a subsequent study by Savian et al. (2018) compared a traditional rotation grazing where animals are moved periodically through different paddocks, with a modified approach where cattle were rotated at a residual forage height of 11 cm, and found that the latter reduced CH₄ production compared to the former. Adding to this body of evidence, DeRamus et al. (2003) observed that rotational grazing resulted in a decrease of about 22% in CH₄ production compared to continuous grazing, while also improving the efficiency of production.

#### 5.1.6 Pros and cons of management strategies

Mitigating CH_4_ emissions through management practices in livestock production offers significant potential for reducing greenhouse gas emissions, but it comes with both advantages and challenges.

The flexibility of these management strategies allows farmers to choose from various options, such as feed additives, dietary adjustments, rumen manipulation, and tailor their approach to specific circumstances. In addition, these methods are effective; for instance, feed additives have shown significant potential, with the ability to reduce CH_4_ emissions in cattle by up to 70% (ERA, 2024) or more (Roque et al., 2021; Kinley et al., 2020). Benchaar et al. (2001) reported that modifying animal diets can lead to up to a 40% reduction in CH_4_ emissions, depending on the specific measures and their extent.

Another significant benefit is the immediate impact of these practices. Unlike long-term strategies, such as selective breeding, the effects of feed additives and dietary changes can be observed quickly, providing rapid progress toward emission reduction goals. Additionally, the use of vaccination in the CH_4_ mitigation strategy has provided a practical and economical alternative (Bezos Earth Fund, 2024b; Baca-González et al., 2020), especially in extensive grazing systems where other mitigation methods may not be feasible. For example, according to Arnesen and Rosenzweig (2025), vaccine production costs are estimated to range from approximately $1 to $4 per dose, which is lower than many feed additives that can cost between $70 to $105 per cow annually. Since vaccines can be administered periodically and during veterinary visits, they could be an economic advantage, thereby minimizing additional labor and logistical costs.

However, these strategies also present several challenges. The ongoing costs of implementing these practices, arising from the continuous use of feed additives and diet manipulation, can be substantial for farmers (Iqbal et al., 2008), especially for smaller operations or in resource-limited areas. In addition, beef producers currently do not get paid or receive any incentives to lower CH_4_ emissions. Moreover, some strategies may have adverse effects on animal health and performance. For instance, the use of fats and oils can negatively impact fiber digestion, potentially delaying the time it takes for an animal to reach finishing weight (McGinn et al., 2004; Jordan et al., 2006). Increasing the concentrate ratio in the feed may also lead to health issues such as acidosis (Iqbal et al., 2008).

Implementation difficulties further complicate the adoption of these practices, particularly in grazing animals. Additionally, certain feed additives, such as ionophores, are antibiotics and face resistance from consumers and regulatory bodies in some countries (Iqbal et al., 2008). Another significant constraint arises from the temporary nature of many interventions. The CH_4_ reducing benefits of treatments like feed additives typically cease once the treatment is discontinued (Ranila et al., 2004). This necessitates continuous application, adding to logistical challenges and ongoing costs.

Despite these challenges, management-based CH_4_ mitigation strategies remain important for reducing CH₄ emissions and addressing climate change. Continued research may improve their cost-effectiveness, practicality, and long-term sustainability. However, because many of these strategies require continuous implementation to maintain their effectiveness, selective breeding has emerged as a long-term approach for reducing CH_4_ emissions in cattle (Lassen & Difford, 2020; de Haas et al., 2021). In practice, sustainable CH_4_ mitigation will likely depend on integrated methods that combine genetic selection with management strategies.

### 5.2 Mitigating methane emissions through selective breeding

Selective breeding in ruminants, including sheep, beef cattle, and dairy cattle, has been shown to reduce CH₄ emissions (Pinares-Patiño et al., 2013; Jonker et al., 2017; de Haas et al., 2021). A major advantage of this approach is its gradual yet cumulative effect, which may contribute to long-term and sustainable CH₄ mitigation. For example, Pinares-Patiño et al. (2011) reported that sheep genetically selected for low CH₄ yield consistently maintained lower CH₄ emissions across different diets and testing periods. Similar results were observed in beef heifers fed forage-based diets (Michal, 2013). Despite its potential, selection for reduced CH₄ emissions may lead to unfavorable correlated responses in economically important traits such as DMI, average daily gain (ADG), and live weight. However, the use of multiple-trait selection indices can support balanced genetic progress by incorporating trait heritability, genetic correlations, and breeding value accuracy (Mrode, 2014). Likewise, de Haas et al. (2021) suggested that assigning economic value to CH₄ emissions within breeding objectives that also include health, fertility, and longevity could support the development of animals that are both lower CH₄ emitters and biologically efficient. Achieving this, however, requires CH₄ phenotypes from large animal populations for accurate genetic evaluation and selection.

#### 5.2.1 Variation in methane emission among animals and heritability

Methane emissions exhibit variability among individual animals and between breeds (Goopy et al., 2011; Pinares-Patiño et al., 2013). Considerable genetic variation in CH₄ emissions has been reported in cattle, indicating potential for selective breeding to reduce emissions (Bell et al., 2014; Lassen & Løvendahl, 2016). Supporting this, Hayes et al. (2016) found that genome-wide SNPs explained 41–67% of the additive genetic variance for CH₄ traits in beef cattle. Likewise, Garnsworthy et al. (2012b) reported significant sire effects on CH₄ emissions, further indicating that CH4 related traits are heritable and suitable for inclusion in breeding programs.

Several studies have estimated the heritability of different CH_4_ related traits in cattle using various measurement technologies. Table 2 summarizes the range of heritability estimates for CH₄ traits, and detailed estimates and corresponding references are provided in Supplementary Table S1. In beef cattle, Donoghue et al. (2013) reported heritability estimates of 0.40 for CH₄ production (g/day) and 0.19 for CH₄ yield. In dairy cattle, heritability estimates for CH₄ production ranged from approximately 0.21 (Lassen et al., 2012; Lassen & Løvendahl, 2016; de Haas et al., 2021) to 0.27 using Fourier-transform infrared spectroscopy (Pszczola et al., 2017). Studies using sniffer systems to measure CH₄ concentration reported heritability estimates ranging from 0.10 to 0.26 (Olijhoek et al., 2020; van Engelen et al., 2018), whereas Pickering et al. (2015a) reported a lower estimate of 0.05 using a Laser Methane Detector. Similarly, Breider et al. (2019) observed heritability estimates ranging from 0.12 to 0.45 for CH₄ production across different stages of lactation in Holstein-Friesian cattle. Differences among studies likely reflect variation in CH_4_ traits evaluated, measurement methods, and study populations. Collectively, these findings indicate that CH_4_ related traits are moderately heritable and suitable for inclusion in cattle breeding programs.

**Table 2:** Summary of reported heritability estimates and correlations between methane traits and economically important traits in cattle.

**Methane trait**	**Heritability range**	**Positively correlated traits**	**Negatively correlated traits**
Methane production (g/d, L/d)	0.09 - 0.46	DMI, live weight, birth weight, weaning weight, yearling weight, ADG, FI, RFI, Longevity	FCR
Methane yield (g/kg DMI)	0.14 - 0.30	ADG	DMI, live weight, ADG, RFI
Methane intensity (g/kg of body weight, g/L of milk)	0.15 - 0.42	FCR, RFI	DMI, live weight, ADG, RFI
Residual methane production (g/d)	0.12 - 0.39	DMI, ADG, RFI	DMI, RFI
Predicted methane emission (g/d)	0.05 - 0.62		DMI, Live weight, RFI, Fertility, Longevity

**DMI = Dry matter intake, ADG = Average daily gain, FI = Feed intake, FCR = Feed conversion ratio, RFI = Residual feed intake**.

**Note:** Heritability estimates and correlated traits were summarized from studies presented in Supplementary Tables S1 and S2.

#### 5.2.2 Genomic selection on methane emissions

Effective genetic selection depends on accurate prediction of an animal’s genetic merit, traditionally based on phenotypic and/or pedigree information. The development of affordable genome-wide DNA marker chips has accelerated the adoption of genomic selection, which uses training populations with both phenotypic and genotypic data to estimate breeding values for selection candidates based solely on DNA marker information (Meuwissen et al., 2001). This method is useful for traits that are difficult or expensive to measure, including CH₄ emission traits. Consequently, genomic selection provides a practical strategy for achieving long-term and cumulative reductions in enteric CH₄ emissions while maintaining productivity within balanced breeding programs. Although implementation may require substantial time, the resulting genetic gains are expected to provide more permanent and sustainable mitigation effects than management-based approaches alone.

The effectiveness of genomic selection is influenced by factors such as trait heritability, the size of the reference population, linkage disequilibrium between genetic markers and quantitative trait loci (QTL), the genetic relationship between reference and selection animals, and the statistical model used for prediction (Goddard & Hayes, 2009; Hayes et al., 2016; Lee et al., 2017; Mrode et al., 2019). For enteric CH₄ emission traits, accurate estimation of genomic breeding values requires large reference populations with both CH₄ phenotypes and genotype data. In addition to improving the accuracy of genetic evaluations, these datasets can be used to identify genomic regions and estimate marker effects associated with CH4 emissions, thereby enhancing prediction of genetic merit for the trait (Calus et al., 2013).

Developing suitable training populations remains a major challenge for implementing genomic selection in beef cattle, particularly because the industry commonly relies on mixed-breed and crossbred populations (Asselstine et al., 2021). Although these populations combine desirable traits from different breeds, their genetic diversity can reduce the reliability of genomic predictions if not properly accounted for. Expanding the reference population can help capture greater genetic diversity and improve genomic prediction performance (Hayes et al., 2009; Meuwissen et al., 2016), which generally increases with larger training datasets (VanRaden et al., 2009). For CH₄ traits with low to moderate heritability, it was estimated that approximately 6000 animals would be required to achieve a prediction accuracy of 0.50 based on the study by Goddard and Hayes (2009), assuming heritability of 0.3 and an effective population size (Ne) of 100. Hayes et al. (2016) reported lower accuracies (0.29–0.35) for CH₄ emissions in 747 Angus cattle, likely reflecting the smaller reference population and population structure.

#### 5.2.3 DNA variants of cattle and variations in rumen microbiomes related to methane emissions

Genome-wide association studies (GWAS) are used to identify DNA variants associated with specific traits, such as CH_4_ related traits, and improve understanding of their genetic architecture, thereby supporting marker-assisted selection and breeding for reduced CH₄ emissions. Several studies have identified SNPs associated with predicted CH_4_ emissions (PME) in cattle, although results have varied across populations and methodologies. For example, Pickering et al. (2015a) identified a SNP on chromosome 7 in dairy cattle associated with PME, with two nearby candidate genes, and Lakamp et al.(2023) reported a significant SNP on the same chromosome in beef cattle, along with 30 SNPs close to the significance threshold. However, Uemoto et al. (2020) found no significant SNPs for CH_4_ emission in beef cattle. Other studies identified associations on different chromosomes: De Haas et al. (2011) reported seven SNPs linked to PME on chromosomes 13, 18, 24, 26, and 27, while Hayes et al. (2016) found the largest-effect SNP for CH_4_ production on chromosome 12, although its probability of a true large effect was only 8%. They also reported a SNP on chromosome 20 linked to CH₄ yield with a 4% probability, while other CH_4_ related traits had approximately a 1.3% probability. These results suggest that CH_4_ emission traits are polygenic, controlled by many genes with small effects rather than a single major gene, making selective breeding more complex but still useful for long-term emission reduction.

Metagenomic analysis involves sequencing and studying the collective genomes of rumen microorganisms to evaluate differences in microbial composition and activity between low- and high CH_4_ emitting cattle. Wallace et al. (2015) reported differences in rumen microbial communities between low and high CH₄ emitters, with archaea more abundant in high-emitting cattle, and Proteobacteria, specifically members of the family *Succinivibrionaceae*, are more prevalent in low emitters. Similarly, Shi et al. (2014) observed a stronger correlation between CH₄ emissions and microbial gene expression (metatranscriptome) than with gene abundance alone, emphasizing the importance of microbial activity in CH₄ production. These findings suggest that manipulation of rumen microbial communities may provide opportunities for CH₄ mitigation. In addition, Hess et al. (2023) demonstrated that integrating rumen metagenomic profiles with host genomic information improved prediction accuracy for CH₄ emissions, feed efficiency, and health traits in livestock.

#### 5.2.4 Potential impacts of genetic selection for reduced methane emissions on economically important and bioindicator traits

Table 2 contains a summary of the relationship between CH_4_ traits and economically important traits in cattle. Detailed estimates and corresponding references are provided in Supplementary Table S2.

##### 5.2.4.1 Dry matter intake (DMI)

A strong positive genetic correlation (r = 0.84 ± 0.06) and moderate to high phenotypic correlation (r = 0.71 ± 0.02) exist between CH₄ emissions and DMI in cattle, indicating that increased feed consumption leads to higher CH₄ production (Donoghue et al., 2016). This relationship is supported by several studies across different cattle breeds and production systems. Pickering et al. (2015a) estimated PME in British dairy cows using the formula developed by de Haas et al. (2011) and found a near-perfect genetic correlation range of 0.99 to 1.00 between PME and DMI. This strong correlation may be attributed to the PME calculation's dependence on the animal's DMI. In Angus beef cattle, research by Donoghue et al. (2016) and Manzanilla-Pech et al. (2016) found a strong genetic relationship between DMI and CH₄ production in beef cattle, with both studies reporting high genetic correlations (r = 0.84 ± 0.06 and 0.83 ± 0.05, respectively) using traditional and genomic best linear unbiased prediction (BLUP) approaches.

The phenotypic correlations between CH₄ production and DMI have shown some variation across studies. Fitzsimons et al. (2013), using Simmental heifers, reported a moderate positive phenotypic correlation of 0.43. In contrast, both Donoghue et al. (2016) and Manzanilla-Pech et al. (2016) found stronger phenotypic correlations of 0.71 (± 0.02) and 0.70 (± 0.02), respectively, in a larger populations of Angus cattle. The discrepancy in these results may be attributed to differences in sample size, with Fitzsimons et al. (2013) using only 22 heifers compared to over 1000 animals in the other studies. These findings suggest that the relationship between DMI and CH₄ production remains relatively stable across beef cattle populations.

The underlying mechanism for this relationship is straightforward: increased feed intake provides more substrate for ruminal fermentation, resulting in higher CH₄ production. However, it is important to note that this correlation can be influenced by factors such as feed composition, breed, age, production stage, and measurement techniques. Nevertheless, the strong correlations of DMI with CH_4_ emissions indicate an opportunity to reduce CH_4_ emissions through genetic selection on DMI when CH₄ emission traits are not available.

##### 5.2.4.2 Live weight

Methane emissions have been observed to increase with rising live weight when animals are fed a maintenance ration (Pickering et al., 2015b). This relationship likely stems from the correlation between live weight and DMI, as larger animals consume more feed, resulting in greater ruminal fermentation and CH₄ production (Manzanilla-Pech et al., 2016). Donoghue et al. (2013) reported a strong positive genetic correlation between CH₄ production and live weight (r = 0.79 ± 0.12), but much weaker relationships for CH₄ yield (r = 0.18 ± 0.30) and CH₄ intensity (r = −0.23 ± 0.28). Similar trends were observed for the corresponding phenotypic correlations of 0.58 ± 0.03, 0.05 ± 0.05, and −0.28 ± 0.05 for CH₄ production, yield, and intensity, respectively. These findings indicate that the relationship between CH₄ emissions and live weight varies depending on the specific CH₄ metric considered.

The correlation between live weight and CH₄ emissions is also influenced by the animal's age. Donoghue et al. (2016) reported that correlations between CH₄ production and body weight strengthened as cattle matured, with genetic correlations increasing from 0.36 ± 0.18 for birth weight to 0.84 ± 0.09 for weaning weight and 0.86 ± 0.06 for yearling weight. Corresponding phenotypic correlations increased from 0.26 ± 0.04 to 0.53 ± 0.03 and 0.61 ± 0.09, respectively. However, the relationship between body weight and CH₄ traits changes as animals age, since both CH₄ production and yield are influenced by the animal’s stage of development and diet composition. Liu et al. (2017) found that heifers produced less total CH₄ but had a higher CH₄ yield compared to mature cows. Similarly, Grandl et al. (2016) observed that CH₄ yield in dairy cows increased with age, peaking at around 2000 days, before gradually declining, showing that physiological development affects CH₄ emissions.

In dairy cattle, Pickering et al. (2015a) found an average genetic correlation of 0.84 ± 0.05 and a phenotypic correlation of 0.49 ± 0.02 between PME and live weight. Interestingly, they observed a gradual decrease in the genetic correlation between PME and live weight during lactation, from weeks 1 to 44, with the correlation dropping from 0.93 in early lactation to 0.62 by the 44th week. Breider et al. (2019) explained this trend by noting that cows typically experience weight loss at the start of lactation due to high energy demands, but as lactation progresses, feed intake peaks around mid-lactation, leading to increased rumen fermentation and higher CH_4_ production.

It is important to note that the correlation between body weight and CH₄ production can vary because, while body weight influences feed consumption, the efficiency of digesting and converting feed to energy (which leads to CH_4_ production) is not solely dependent on body weight. Other factors, such as diet composition, individual animal genetics, and rumen microbial population, also play significant roles in determining CH₄ emissions.

##### 5.2.4.3 Feed efficiency measures

Residual Feed Intake (RFI) is an important indicator of feed efficiency in cattle. It is independent of animal growth and body weight as it is estimated by the difference between observed and expected feed intake given the animal growth rate and body weight. Studies focusing on RFI have found consistent moderate phenotypic correlations between RFI and CH₄ production. Nkrumah et al. (2006), McDonnell et al. (2016) and Velazco et al. (2017) reported similar correlations of 0.38, 0.42, and 0.41 (all *P* < 0.05), respectively, when selecting animals based on RFI differences. Improving feed utilization efficiency through lower RFI values has the potential to significantly reduce CH₄ emissions, as more efficient animals typically produce less CH₄ per unit of feed consumed (Potts et al., 2015). Fitzsimons et al. (2013) reported that a reduction of 1 kg dry matter per day in RFI can lead to a 23 g/d decrease in CH₄ emission, highlighting the potential impact of RFI selection on CH_4_ mitigation strategies.

The relationship between CH₄ emissions and RFI also shows variability across studies. Fitzsimons et al. (2013) reported positive correlations between RFI and CH_4_ production (0.26), and CH₄ intensity (0.30), indicating that more efficient animals (lower RFI) emit less CH₄. This finding is supported by Hegarty et al. (2007) and Manafiazar et al. (2020), who observed lower CH₄ production in cattle with low RFI compared to those with high RFI. However, other studies have found conflicting results. McDonnell et al. (2016) and Jones et al. (2011) did not find a correlation between RFI and CH₄ production. Some researchers even found negative correlations between RFI and CH₄ traits. Herd et al. (2016) noted a correlation of −0.37, while Fitzsimons et al. (2013) found a correlation of −0.27 with CH₄ yield, and Velazco et al. (2017) reported a negative correlation of −0.55 with PME.

These conflicting results highlight the complex relationship between feed efficiency measures and CH₄ emissions. Although RFI is linked to feed intake, a primary driver of CH₄ production, other factors such as diet composition, rumen microbial populations, and individual animal genetics can influence CH_4_ emissions. Variability in findings across studies can also be attributed to differences in experimental conditions, animal populations, measurement techniques, and the specific efficiency metrics employed.

Variation in RFI estimates among animals shows variability in metabolic responses, and according to Richardson and Herd (2004) the metabolic response comprises about two-thirds of the total RFI variation. This makes RFI a useful trait to select for improved herd feed efficiency. On the other hand, feed conversion ratio (FCR) is dependent on animal growth and body weight. FCR changes directly with body weight, as it is estimated as the ratio of feed intake against weight gain. Selecting an animal for improved FCR can lead to an increase in growth rate and body weight, making it a useful trait to enhance growth efficiency. Herd et al. (2016) found a negative phenotypic correlation of −0.31 between FCR and CH₄ production in beef cattle fed roughages, which is an indication that animals that have a lower FCR might produce more CH₄. Interestingly, this correlation was not observed in those fed concentrates. Similarly, Velazco et al. (2017) found no correlation between FCR and CH₄ production in beef cattle grazing on pasture and paddocks. Durunna et al. (2012) found that reranking in RFI occurred at different periods in heifers placed on the same diet, containing 10% barley grain and 90% barley silage, with about 51% of the heifers changing their RFI class. This suggests that RFI can change over time in animals, despite being fed the same diets.

##### 5.2.4.4 Fertility traits

Fertility traits are crucial indicators of reproductive performance and breeding success in livestock. Research has revealed a complex relationship between these traits and CH₄ emissions in cattle. Kandel et al. (2018) reported a low but positive genetic correlation between CH₄ emissions and fertility traits such as direct calving ease, maternal calving ease, and pregnancy rate. This suggests that animals emitting more CH₄ often exhibit better fertility, presenting a potential trade-off in breeding programs aimed at reducing GHG emissions. However, the relationship between fertility and overall herd CH₄ emissions is multifaceted. Knapp et al. (2014) noted that improving fertility traits can lead to a reduction in total herd CH₄ emissions. This is because enhanced fertility reduces the need for replacement heifers, resulting in a more efficient herd with fewer animals producing more offspring.

Zetouni et al. (2018) provided insights into the genetic correlations between CH₄ production and certain fertility traits. They observed low to moderate correlations of 0.28 (±0.21) for the first to last insemination interval, 0.17 (±0.13) for the calving to first insemination interval, and 0.07 (±0.17) for the number of inseminations with total CH_4_ emission. These findings suggest that animals with shorter fertility-related intervals and fewer fertility issues tend to produce less CH_4_. Importantly, these genetic correlations, while positive, are relatively low. This indicates that selecting for reduced CH₄ emissions is unlikely to significantly impair fertility traits. On the other hand, it also suggests that improving fertility alone may not substantially reduce CH₄ emissions at the individual animal level.

##### 5.2.4.5 Longevity

Kandel et al. (2018) observed a positive genetic correlation (r = 0.22) between CH₄ emissions predicted from the milk of dairy cows and breeding values of sire longevity in cattle. This suggests that animals with high CH_4_ emissions tend to have longer productive lives. The study analyzed records from 58,412 cows in their first three parities, sired by 2455 bulls, accounting for multiple economically important traits, including CH₄ emissions and longevity. Paradoxically, enhanced longevity can contribute to reduced herd-level CH_4_ emissions by decreasing the need for replacement heifers. However, Pszczola et al. (2019) found a low negative correlation of −0.06 (±0.07) between CH₄ production and longevity. This contrasting finding suggests that strategies aimed at improving longevity might also have the potential to lower CH₄ production, although the relationship appears weak.

#### 5.2.5 Potential impacts of genetic selection for reduced methane emissions on bioindicator traits

Potential bioindicators of CH₄ emissions, such as milk composition, rumen microbiome, and fecal methanogens, have shown significant correlations with CH₄ emissions, making them valuable tools for researchers and breeders to select cattle with reduced CH₄ emissions. However, some bioindicator traits are also economically relevant, such as milk composition, or are related to animal health/well-being, such as rumen microbiome, and fecal methanogens. Studies have demonstrated these correlations: milk fatty acids (r = −0.56 – 0.42) with CH₄ yield (g/kg; van Lingen et al., 2014), fecal methanogens (r = 0.53) with CH₄ production (g/d; Manafiazar et al., 2021), and rumen microbial profiles (r = 0.49) with CH_4_ yield (g/DMI; Wallace et al., 2014), rumen volatile fatty acid (r = 0.40) with CH₄ yield (L/kg DMI), and with CH_4_ intensity (r = 0.40, L/kg weight) by Herd et al. (2013). These proxies offer cost-effective, scalable, and less invasive methods for estimating CH_4_ production, making them highly useful for large-scale breeding programs aimed at reducing emissions. However, selection on the bioindicator traits to reduce CH₄ emissions will also lead to changes in the bioindicator traits themselves.

#### 5.2.6 Pros and cons of selective breeding

Selective breeding offers a sustainable, long-term solution for reducing CH₄ emissions, and its results accumulate over generations, leading to a progressive reduction in CH₄ emissions (Wall et al., 2010; de Haas et al., 2021). The accumulated reduction remains permanent; an animal that is bred for low CH_4_ emission passes this trait or genes to its offspring, and that continues. Once implemented, genetic improvements incur no ongoing costs, unlike feed additives or management changes that require continuous application, making it a cost-effective strategy. This method can be integrated with other mitigation strategies, potentially enhancing their effectiveness and leading to a more comprehensive approach to reducing CH₄ emissions.

Breeding for lower CH₄ emissions often correlates with improved feed efficiency, resulting in economic benefits for farmers through reduced feed costs and enhanced productivity (Basarab et al., 2013). Genomic selection allows for more accurate and rapid genetic improvement compared to traditional breeding methods, accelerating the achievement of desired traits (Hayes et al., 2013). Genomic selection can be implemented based on direct CH4 records and/or proxy traits and a recent national validation demonstrated that genomic breeding values can successfully identify animals with lower CH4 emissions (Narayana et al., 2026).

Despite its potential, selective breeding faces several challenges. Genetic improvements take time to manifest across populations, typically requiring generations to yield significant results, making it a long-term investment (Wall et al., 2010). Methane emission is a polygenic trait influenced by multiple genes, which complicates the selection process due to its complex genetic basis (de Haas et al., 2011; Hayes et al., 2016). Additionally, accurately measuring individual animal CH₄ emissions for genetic evaluation or for development of genomic selection reference population can be difficult and expensive, posing a significant challenge to the widespread adoption of this approach (Negussie et al., 2017). National and international collaborations to consolidate CH_4_ emissions data will help improve the accuracy of genetic evaluation or genomic selection on CH_4_ emissions.

Selective breeding to reduce CH₄ emissions can also be conducted on alternative traits and/or bioindicator traits. Amer (2018) reported that genetic improvements on production, fertility and survival traits in the Irish dairy industry reduced emission intensities by ∼5% in the last 10 years. When genetic evaluation on CH₄ emission traits is available, CH₄ emissions can be included as a new trait in breeding programs for direct selection (de Haas et al., 2021). However, either direct selection or indirect selection has a risk that selecting for reduced CH₄ emissions may inadvertently affect other important traits if not carefully managed, necessitating a balanced approach to breeding (Bird-Gardiner et al., 2015; Donoghue et al., 2016). These issues can be mitigated by simultaneously integrating CH4 traits and other economically relevant traits into the breeding objective. According to Shi et al. (2024), breeding programs aimed at reducing GHG emissions can utilize two distinct methods. One method involves assigning economic value to CH₄ emissions and integrating it into a multiple-trait selection or breeding index to introduce a new trait into the breeding goal. The second method, however, focuses on leveraging the impact of existing heritable traits, including the bioindicators and environmentally beneficial trait combinations within the selection or breeding index, to reduce CH₄ emissions without introducing entirely new traits.

## 6 Current national-global initiative, research consortium to reduce methane-greenhouse gas production by cattle

The European Union and the United States initiated the Global Methane Pledge (GMP), a significant international effort to reduce CH₄ emissions across various sectors, such as the agricultural industry (GMP, 2024). The Global Methane Pledge establishes a challenging and collective objective to decrease global CH₄ emissions by a minimum of 30% by 2030, with 2020 emission levels serving as the reference point, and as of now, over 155 countries have joined this initiative. In support of this global effort, several key initiatives have been established. The Greener Cattle Initiative, founded by the Foundation for Food & Agriculture Research (FFAR), brings together US and international stakeholders to invest in research aimed at reducing CH₄ emissions from both dairy and beef cattle (FFAR, 2023). This initiative focuses on understanding the impact of CH₄ mitigation strategies on cattle performance and exploring selective breeding and new approaches to reduce emissions. By promoting collaboration between industry, academia, and government, the Greener Cattle Initiative aims to accelerate the development of practical solutions for the livestock sector. As of early 2024, the Greener Cattle Initiative is set to award more than $5 million in grants to support research aimed at reducing enteric CH₄ emissions in dairy and beef cattle. The program has received industry backing and submissions from academic and research institutions, with many proposals currently under active review for funding and implementation.

Similarly, the Global Methane Hub, an international organization established in 2021, fosters collaboration between stakeholders, organizations, investors, researchers, and governments to reduce CH₄ emissions from the agricultural, waste, and energy sectors (Global Methane Hub, 2024). In collaboration with The Bezos Earth Fund, Global Methane Hub launched the Global Methane Genetics Initiative, a program aimed at reducing CH₄ emissions through genetic improvement by breeding for low-CH₄ animals (Bezos Earth Fund, 2024a). The initiative promotes large-scale data sharing, standardized CH₄ measurement approaches, and collaborative research to facilitate the inclusion of CH_4_ related traits in national breeding programs.

The Animal Selection, Genetics and Genomics Network (ASGGN), is an international network that operates within the Livestock Research Group of the Global Research Alliance on Agricultural Greenhouse Gases. The network brings together researchers from multiple countries to improve the consistency of CH₄ phenotyping, encourage data sharing, standardize DNA and rumen sample collection, and support joint analyses (ASGGN, 2014). These collaborative efforts are intended to facilitate the incorporation of CH4 related traits into livestock breeding programs and accelerate genetic progress toward lower-emitting animals.

In Ireland, Teagasc, together with University College Dublin and the Agri-Food and Biosciences Institute, established the RU-MIT-LESS project to evaluate the effects of feed additives designed to reduce CH_4_ emissions in beef cattle under pasture and indoor management systems (Teagasc, 2025). The project also evaluates wearable CH_4_ sensors, animal productivity, economic performance, and environmental outcomes to provide a comprehensive assessment of mitigation strategies. Similarly, Meat & Livestock Australia (MLA) has also made CH_4_ reduction an important part of its Strategic Plan 2030, which aims to improve industry productivity, profitability, and sustainability (MLA, 2025). To achieve CH_4_ reduction, MLA is investing in CH4 genetics, feed additives, pasture management, and the development of practical and affordable CH_4_ and soil carbon measurement technologies (MLA, 2025).

Several countries are adopting policy and management strategies to reduce CH_4_ emissions from livestock production; Denmark has introduced the world's first GHG tax on livestock emissions, which will take effect in 2030 (Reuters, 2024). The policy applies a tax based on the CO₂ equivalent of emissions produced by livestock and is intended to encourage producers to implement CH_4_ mitigation practices and transition to more sustainable production systems. The National Institute of Agricultural Research (INIA) in Uruguay has joined the Global Methane Genetics Initiative to strengthen international collaboration on CH_4_ phenotyping and genetic improvement (INIA, 2025).

Canada has taken a proactive stance in addressing CH₄ emissions from agriculture to foster the Global Methane Pledge 2030. In 2023, the Canadian government introduced the Agricultural Methane Reduction Challenge, offering $12 million in prizes to encourage innovative solutions for reducing CH₄ emissions from cattle across various sectors (Agriculture & Agri-Food Canada, 2023). This challenge aims to stimulate the development of inventive and adaptable strategies for mitigating CH₄ emissions in the cow-calf sector, feedlots, and dairy farms. Additionally, the Canadian government launched the Reducing Enteric Methane Emission from Beef Cattle (REME) program (Environment & Climate Change Canada, 2023), allowing beef cattle farmers to earn compensation credits for implementing CH4 reducing strategies. These credits represent quantifiable reductions in enteric CH₄ emissions achieved through improved cattle management, diet modifications, breeding, and other approaches. More information, including program details and eligibility, is available at the official Government of Canada REME program page.

Genetic approaches are also being actively explored as a promising avenue for CH₄ reduction. Companies like Semex are collaborating with Lactanet, a Canadian milk-recording and dairy advisory agency, to produce and market low CH4 breeding material for dairy cattle (Nickel, 2023). The local effort and international collaborations offer opportunities for consolidation of a larger CH₄ emission reference data set for genomic selection. In April 2023, Lactanet released the world's first national genomic evaluation of CH₄, covering about 6000 farms. This initiative has led to the adoption of climate-friendly breeding practices by farmers. These efforts demonstrate the potential for genetic selection to play a significant role in reducing livestock CH₄ emissions.

Lactanet and PLQ (Les Producteurs de lait du Québec) are also leading a research project from July 2023 to 2027, to estimate enteric CH_4_ emissions on commercial dairy farms using infrared spectra from routine bulk tank milk samples, combined with sensor data and farm production records (Lactanet, 2023). The project’s first phase focuses on developing an algorithm that links CH_4_ to the milk infrared reflectance spectrum and other economically important traits. The second phase of the project will involve a large-scale validation across 50 herds representing various management, genetics, and feeding systems. This will support the implementation of CH₄ mitigation strategies on the selected farms in the final stages of the project. The project also aims to estimate emissions from all cattle groups, assess other farm-related GHG sources, and evaluate mitigation strategies and their economic impacts. Ultimately, the initiative will improve genetic selection for CH₄ efficiency and set up an online system so farmers can easily access CH₄ estimates for their cows using the results from bulk tank milk tests.

Climate Action Through Grazing (CAT-G) is a research initiative in Canada that is aimed at improving carbon sequestration in grasslands, reducing CH₄ emissions, and improving productivity in cattle (CAT-G Project, 2023). The research is led by researchers from the University of Alberta in partnership with Agriculture and Agri-Food Canada and working closely with producers and industry stakeholders (CAT-G project, 2023). This project applies genomic approaches to examine how grazing management affects both soil and bovine fecal microbiomes (Genome Alberta, 2023). It aims to identify biological indicators and key mechanisms associated with enhanced soil carbon sequestration and reduced greenhouse gas emissions across soils, plants, and grazing cattle. The resulting datasets will support the development of bio-indicator tools that reflect carbon cycling dynamics in grassland systems (Genome Alberta, 2023 ).

Innovative feed additives are another area of active research and development. The "Project Clean Cow," a partnership between Royal DSM, other companies, and universities in Canada, has developed a promising feed additive, 3-NOP (ERA, 2024). Partly funded by Emissions Reduction Alberta (ERA), this additive modifies the animal's digestive system to reduce CH₄ emissions without adversely affecting health or production. Large-scale demonstrations have shown potential for up to 70% reduction in enteric CH₄ emissions, significantly exceeding the reductions of 30% observed in smaller trials (ERA, 2024). This breakthrough points out the possibility for technological solutions to make a substantial impact on livestock CH_4_ emissions. The above various initiatives show the increasing global commitment to tackling CH₄ emissions from cattle, integrating technological innovations, genetic strategies, and policy measures to achieve significant reductions of CH₄ emissions in line with climate change mitigation goals.

## 7 Future directions in methane emission reduction

Future CH₄ mitigation research is likely to rely more on precision livestock systems, expanded CH_4_ phenotyping, genomic selection, rumen microbiome characterization, and artificial intelligence-driven predictive models. Advances in sensor technologies and automated data collection systems may improve the feasibility of recording CH₄ emissions in large commercial cattle populations, thereby strengthening genetic evaluation and selection programs. International collaboration, data sharing, and the standardization of CH₄ measurement protocols will also be important for accelerating genetic progress and developing practical long-term CH₄ mitigation strategies.

Combining management practices with selective breeding will likely provide a more effective and holistic CH₄ mitigation strategy than relying on either approach alone. Greater reductions in CH₄ emissions may be achieved when animals genetically selected for lower CH₄ emissions are managed using nutritional interventions, antimethanogenic feed additives, or grazing systems designed to reduce CH₄ production. However, the extent to which different mitigation strategies produce additive, synergistic, or antagonistic effects remains insufficiently understood. In addition, genotype × environment (G × E) interactions may influence the effectiveness of CH₄ mitigation strategies across diets, production systems, and environmental conditions. Consequently, further research is needed to evaluate how breeding and management strategies interact under commercial production settings and how these strategies can be optimally integrated into sustainable cattle production systems. In addition to scientific advances, broader adoption of CH₄ mitigation strategies in commercial beef production systems will likely depend on economic feasibility, producer acceptance, and policy support. Incentive programs such as subsidies, carbon credit systems, sustainability-based policies, and industry-supported extension initiatives may encourage the implementation of CH₄ mitigation strategies and management practices.

## 8 Conclusion

The review highlights the significant impact of CH₄ emissions in beef cattle as a contributor to GHG, emphasizing the need for effective mitigation strategies. Various factors, including diet, animal management practices, and animal genetics, influence CH₄ production. Mitigating CH₄ emissions in cattle through management practices such as feed additives, diet modification, use of vaccinations, and grazing management can lead to immediate reduction of footprints associated with cattle production, while selective breeding for reduced CH₄ emissions in cattle presents a promising long-term strategy for mitigating the environmental impact of cattle production due to its permanent and accumulative benefits. The moderate heritability of CH₄-related traits and their genetic correlations with economically important traits suggest that selective breeding can simultaneously improve production efficiency and reduce emissions through balanced programs. However, challenges remain, particularly in accurately measuring CH₄ emissions and integrating these traits into existing breeding programs. Future efforts should focus on refining measurement techniques, developing cost-effective proxies for large-scale phenotyping, and exploring the potential of technologies like genomic selection. Ultimately, a strategic combination of management practices and selective breeding represents the most promising approach to effectively mitigate CH₄ emissions in cattle, leading to improved economic and environmental outcomes of beef cattle production.

## Declaration-Statement for studies in humans or animals

The study does not use animals or humans or their samples.

## Funding

The authors acknowledge funding and support from Genome Canada and Genome Alberta (Grant #C22GRS); the Governments of Canada and Alberta through the Sustainable Canadian Agricultural Partnership, delivered by Results Driven Agriculture Research (Grant #2023G1491R); the Governments of Canada and Saskatchewan through the Sustainable Canadian Agricultural Partnership and the Agriculture Development Fund (Grant #20240659); and the University of Alberta (Grants #RES0066503, RES0066504, RES0066506). The funding bodies played no role in the design of the study and collection, analysis, and interpretation of data and in writing the manuscript.

## CRediT authorship contribution statement

**Esther Taiwo:** Writing – original draft, Investigation, Formal analysis, Data curation. **Gleise M. Silva:** Writing – review & editing, Formal analysis. **Ghader Manafiazar:** Writing – review & editing, Formal analysis. **Carolyn Fitzsimmons:** Writing – review & editing, Funding acquisition, Formal analysis. **Changxi Li:** Writing – review & editing, Supervision, Formal analysis, Conceptualization.

## Declaration of competing interest

Authors declare that there is no conflict of interest.

## Appendix Supplementary materials

Image, application 1
