# Supplementary material for: Methane emissions in beef cattle: A review of measurements and mitigation strategies
Source: Vet Anim Sci. 2026 Aug 4;33:100801. doi: 10.1016/j.vas.2026.100801 (PMC13524546; doi:10.1016/j.vas.2026.100801)
Supplement: Supplementary file 1 [file mmc1.docx]

**Supplementary Table S1: Heritability estimates of methane emission traits in cattle**

| **Trait** | **Method** | **Number of animals** | **Heritability (SE)** | **Animal**  **Type** | **Reference** |
| --- | --- | --- | --- | --- | --- |
| **MP** | GEM | 200 | 0.46 (014) | Dairy | Layton et al. (2025) |
|  | SF_6_ tracer | 1153 | 0.25 | Beef | Silva et al. (2025) |
|  | GEM | 656 | 0.36 (0.12) | Dairy | Lopes et al. (2024) |
|  | GEM | 1155 | 0.09 – 0.43 | Beef | Ryan et al. (2024) |
|  | GEM | 330 | 0.16 (0.10) | Dairy | Kamalanathan et al. (2023) |
|  | GEM | 979 | 0.31 (0.136) | Beef | Ryan et al. (2022) |
|  | Infrared sensor (AMS) | 575 | 0.21 (0.05) | Dairy | Manzanilla-Pech et al. (2022) |
|  | Sniffer (milking robot) |  | 0.21 | Dairy | de Haas et al. (2021) |
|  | RC | 1043 | 0.24 (0.06) | Beef | Arthur et al. (2018) |
|  | Meta-analysis |  | 0.22-0.29 | Both | Brito et al. (2018) |
|  | FTIR (AMS) | 1745 | 0.21 (0.06) | Dairy | Lassen & Løvendahl (2016) |
|  | Respiratory Chamber | 1043 | 0.28 (0.06) | Beef | Hayes et al. (2016) |
|  |  | 1020 | 0.30 (0.06) | Beef | Manzanilla-Pech et al. (2016) |
|  |  | 903 | 0.23 | Dairy | Manzanilla-Pech et al. (2016) |
|  | RC | 530 | 0.21 (0.11) | Beef | Donoghue et al. (2013) |
| **MY** | SF_6_ tracer | 1153 | 0.14 | Beef | Silva et al. (2025) |
|  | GEM | 287 | 0.27 (0.12) | Dairy | Kamalanathan et al. (2023) |
|  |  | 572 | 0.22 (0.05) | Dairy | Manzanilla-Pech et al. (2022) |
|  | Respiratory Chamber | 1043 | 0.20 (0.05) | Beef | Hayes et al. (2016) |
|  | RC | 1043 | 0.22 (0.06) | Beef | Arthur et al. (2018) |
|  | Meta-analysis |  | 0.16–0.29 | Cattle | Brito et al. (2018) |
|  |  | 1020 | 0.20 (0.05) | Beef | Manzanilla-Pech et al. (2016) |
|  |  | 903 | 0.30 | Dairy | Manzanilla-Pech et al. (2016) |
|  | RC | 530 | 0.19 (0.10) | Beef | Donoghue et al. (2013) |
| **MI** | GEM | 265 | 0.21 (0.14) | Dairy | Kamalanathan et al. (2023) |
|  |  | 573 | 0.18 (0.04) | Dairy | Manzanilla-Pech et al. (2022) |
|  | Meta-analysis |  | 0.15-0.23 | Beef and dairy | Brito et al. (2018) |
|  |  | 1020 | 0.25 (0.06) | Beef | Manzanilla-Pech et al. (2016) |
|  |  | 903 | 0.42 | Dairy | Manzanilla-Pech et al. (2016) |
|  |  | 1745 | 0.21 (0.06) | Dairy | Lassen & Løvendahl (2016) |
|  | RC | 530 | 0.23 (0.10) | Beef | Donoghue et al. (2013) |
| **PME** |  | 830 | 0.59 – 0.62 (0.13) | Beef | Lakamp et al. (2023) |
|  | Prediction equation | 1305 | 0.05 – 0.30 | Dairy | Pickering et al. (2015a) |
|  | Predicted equation | 665 | 0.35 | Dairy | De Haas et al. (2011) |
| **RMP** | SF_6_ tracer | 1153 | 0.14 | Beef | Silva et al. (2025) |
|  | GEM | 979 | 0.39 (0.143) | Beef | Ryan et al. (2022) |
|  |  | 511 | 0.16 (0.02) | Dairy | Manzanilla-Pech et al. (2022) |
|  | RC | 1043 | 0.18 (0.06) | Beef | Arthur et al. (2018) |
|  | Meta-analysis |  | 0.12–0.22 | Beef and dairy | Brito et al. (2018) |
|  | Respiratory Chamber | 1043 | 0.18 (0.05) | Beef | Hayes et al. (2016) |

SE = Standard Error, MP = Methane production, MI = Methane intensity, MY = Methane yield, PME = Predicted methane production, RMP = Residual methane production, RC = Respiratory chamber, FTIR = Fourier, GEM = GreenFeed® emissions, AMS = Automated milking system

**Supplementary Table S2: Genetic and phenotypic correlations between methane traits and economically important traits in cattle**

| **Methane Traits** | **Economically Important Traits** | **Correlation coefficient r (SE)** | **Type of Correlation** | **Type of animal** | | **References** |
| --- | --- | --- | --- | --- | --- | --- |
| MP | Live weight | 0.430(0.047) | Phenotypic | Dairy | Layton et al. (2025) | |
|  | Liveweight | 0.177(0.532) | Genetic | Dairy | Layton et al. (2025) | |
|  | Live weight | 0.30 | Phenotypic | Beef | Ryan et al. (2022) | |
| MP | Live weight | 0.68 – 0.70 | Phenotypic | Beef | Renand et al. (2019) | |
|  | Live weight | 0.80 (0.06) | Genetic | Beef | Manzanilla-Pech et al. (2016) | |
|  | Live weight | 0.67 (0.02) | Phenotypic | Beef | Manzanilla-Pech et al. (2016) | |
|  | Birth weight | 0.26 (0.04) | Phenotypic | Beef | Donoghue et al. (2016) | |
|  | Birth weight | 0.36 (0.18) | Genetic | Beef | Donoghue et al. (2016) | |
|  | Weaning weight | 0.53 (0.03) | Phenotypic | Beef | Donoghue et al. (2016) | |
|  | Weaning weight | 0.84 (0.09) | Genetic | Beef | Donoghue et al. (2016) | |
|  | Yearling weight | 0.61 (0.09) | Phenotypic | Beef | Donoghue et al. (2016) | |
|  | Yearling weight | 0.86 (0.06) | Genetic | Beef | Donoghue et al. (2016) | |
|  | ADG | 0.39 (0.11) | Genetic | Beef | Crowley et al. (2026) | |
|  | ADG | 0.30 | Phenotypic | Beef | Crowley et al. (2026) | |
|  | ADG | 0.90 (0.09) | Genetic | Beef | Silva et al. (2025) | |
|  | ADG | 0.26 (0.03) | Phenotypic | Beef | Silva et al. (2025) | |
|  | ADG | 0.26 – 0.44 | Phenotypic | Beef | Renand et al. (2019) | |
|  | Feed intake | 0.549 (0.03) | Phenotypic | Dairy | (Layton et al., 2025) | |
|  | Feed intake | 0.324 (0.55) | Genetic | Dairy | (Layton et al., 2025) | |
|  | Feed intake | 0.49 – 0.76 | Genetic | Beef | (Ryan et al., 2025) | |
|  | DMI | 0.79 (0.10) | Genetic | Beef | Silva et al. (2025) | |
|  | DMI | 0.35 (0.02) | Phenotypic | Beef | Silva et al. (2025) | |
|  | DMI | 0.43 – 0.72 | Genetic | Beef | Ryan et al. (2024) | |
|  | DMI | 0.36 – 0.48 | Phenotypic | Beef | Renand et al. (2019) | |
|  | FCR | -0.17 | Phenotypic | Beef | Velazco et al. (2017) | |
|  | FCR | -0.31 | Phenotypic | Beef | Herd et al. (2016) | |
|  | RFI | 0.27 (0.15) | Genetic | Beef | Silva et al. (2025) | |
|  | RFI | 0.10 (0.26) | Phenotypic | Beef | Silva et al. (2025) | |
|  | RFI | 0.26 | Phenotypic | Beef | Fitzsimons et al. (2013) | |
|  | Longevity | -0.06 (0.07) | Genetic | Dairy | Pszczola et al. (2019) | |
| MY | Live weight | 0.02 - 0.04 | Phenotypic | Beef | Renand et al. (2019) | |
|  | Live weight | 0.18 (0.30) | Genetic | Beef | Donoghue et al. (2013) | |
|  | Live weight | 0.05 (0.05) | Phenotypic | Beef | Donoghue et al. (2013) | |
|  | DMI | -0.07 (0.15) | Genetic | Beef | Silva et al. (2025) | |
|  | DMI | -0.23 (0.03) | Phenotypic | Beef | Silva et al. (2025) | |
|  | DMI | -0.67 - 0.77 | Phenotypic | Beef | Renand et al. (2019) | |
|  | DMI | -0.22 | Phenotypic | Beef | Mc Court et al. (2006) | |
|  | ADG | 0.24 (0.16) | Genotypic | Beef | Silva et al. (2025) | |
|  | ADG | -0.04 (0.03) | Phenotypic | Beef | Silva et al. (2025) | |
|  | ADG | -0.08 | Phenotypic | Beef | Renand et al. (2019) | |
|  | RFI | -0.43 (0.21) | Genetic | Beef | Silva et al. (2025) | |
|  | RFI | -0.32 (0.03) | Phenotypic | Beef | Silva et al. (2025) | |
|  | RFI | -0.37 | Phenotypic | Beef | Herd et al. (2016) | |
|  | RFI | -0.27 | Phenotypic | Beef | Fitzsimons et al. (2013) | |
| MI | Live weight | -0.23 (0.28) | Genetic | Beef | Donoghue et al. (2013) | |
|  | Live weight | -0.28 (0.05) | Phenotypic | Beef | Donoghue et al. (2013) | |
|  | ADG | -0.82 | Phenotypic | Beef | Velazco et al. (2017) | |
|  | DMI | -0.68 | Phenotypic | Beef | Velazco et al. (2017) | |
|  | FCR | 0.97 | Phenotypic | Beef | Velazco et al. (2017) | |
|  | RFI | 0.30 | Phenotypic | Beef | Fitzsimons et al. (2013) | |
| PME | Live weight | 0.22 | Genetic | Dairy | Kandel et al. (2018) | |
|  | Live weight | 0.84 (0.05) | Genetic | Dairy | Pickering et al. (2015a) | |
|  | Live weight | 0.49 | Phenotypic | Dairy | Pickering et al. (2015a) | |
|  | DMI | 0.99 | Phenotypic | Dairy | De Haas et al. (2011) | |
|  | RFI | 0.72 | Phenotypic | Dairy | De Haas et al. (2011) | |
|  | Fertility | 0.17 | Genetic | Dairy | Kandel et al. (2018) | |
|  | longevity | 0.22 | Genetic | Dairy | Kandel et al. (2018) | |
| RME | DMI | 0.19 (0.17) | Genetic | Beef | Silva et al. (2025) | |
|  | DMI | -0.07 (0.03) | Phenotypic | Beef | Silva et al. (2025) | |
|  | ADG | 0.50 (0.21) | Genetic | Beef | Silva et al. (2025) | |
|  | ADG | 0.04 (0.03) | Phenotypic | Beef | Silva et al. (2025) | |
|  | RFI | -0.07 (0.26) | Genetic | Beef | Silva et al. (2025) | |
|  | RFI | 0.18 (0.03) | Phenotypic | Beef | Silva et al. (2025) | |

SE = Standard Error, MP = Methane production, MI = Methane intensity, MY = Methane yield, PME = Predicted methane production, RMP = Residual methane production, RC = Respiratory chamber, FTIR = Fourier, GEM = GreenFeed® emissions, AMS = Automated milking system, DMI = Dry matter intake, ADG = Average daily gain, FCR = Feed conversion ratio, RFI = Residual feed intake

**References**

Arthur, P. F., Donoghue, K. A., Bird-Gardiner, T., Herd, R. M., & Hegarty, R. S. (2018). Effect of measurement duration in respiration chambers on methane traits of beef cattle. Animal Production Science, 58(6), 1006–1010. <https://doi.org/10.1071/AN15425>

Brito L. F., Schenkel F. S., Oliveira H. R., Cánovas A., & Miglior F. (2018). Meta-analysis of heritability estimates for methane emission indicator traits in cattle and sheep. In Proceedings of the 11th World Congress on Genetics Applied to Livestock Production, Auckland, New Zealand, 7–11.

Crowley, S. B., Purfield, D. C., Conroy, S., Kelly, D. N., Evans, R. D., Ryan, C. V., & Berry, D. P. (2026). Genetic insights into enteric methane emissions in indoor-fed growing cattle. Journal of Animal Science, 104, skag046. <https://doi.org/10.1093/jas/skag046>

Donoghue, K. A., Bird-Gardiner, T., Arthur, P. F., Herd, R. M., & Hegarty, R. F. (2016). Genetic and phenotypic variance and covariance components for methane emission and postweaning traits in Angus cattle12. Journal of Animal Science, 94(4), 1438–1445. <https://doi.org/10.2527/jas.2015-0065>

Donoghue, K. A., Herd, R. M., Bird, S. H., Arthur, P. F., & Hegarty, R. S. (2013). Preliminary genetic parameters for methane production in Australian beef cattle. In Proceedings of the 20th Conference of the Association for the Advancement of Animal Breeding and Genetics (pp. 290–293).

de Haas, Y., Veerkamp, R. F., de Jong, G., & Aldridge, M. N. (2021). Selective breeding as a mitigation tool for methane emissions from dairy cattle. Animal, 15, 100294. <https://doi.org/10.1016/j.animal.2021.100294>

De Haas, Y., Windig, J. J., Calus, M. P. L., Dijkstra, J., de Haan, M., Bannink, A., & Veerkamp, R. F. (2011). Genetic parameters for predicted methane production and potential for reducing enteric emissions through genomic selection. Journal of Dairy Science, 94(12), 6122–6134. <https://doi.org/10.3168/jds.2011-4439>

Fitzsimons, C., Kenny, D. A., Deighton, M. H., Fahey, A. G., & McGee, M. (2013). Methane emissions, body composition, and rumen fermentation traits of beef heifers differing in residual feed intake1. Journal of Animal Science, 91(12), 5789–5800. <https://doi.org/10.2527/jas.2013-6956>

Hayes, B. J., Donoghue, K. A., Reich, C. M., Mason, B. A., Bird-Gardiner, T., Herd, R. M., & Arthur, P. F. (2016). Genomic heritabilities and genomic estimated breeding values for methane traits in Angus cattle1. Journal of Animal Science, 94(3), 902–908. <https://doi.org/10.2527/jas.2015-0078>

Herd, R. M., Velazco, J. I., Arthur, P. F., & Hegarty, R. F. (2016). Associations among methane emission traits measured in the feedlot and in respiration chambers in Angus cattle bred to vary in feed efficiency1. Journal of Animal Science, 94(11), 4882–4891. <https://doi.org/10.2527/jas.2016-0613>

Kamalanathan, S., Houlahan, K., Miglior, F., Chud, T. C. S., Seymour, D. J., Hailemariam, D., Plastow, G., de Oliveira, H. R., Baes, C. F., & Schenkel, F. S. (2023). Genetic Analysis of Methane Emission Traits in Holstein Dairy Cattle. Animals, 13(8), 1308. <https://doi.org/10.3390/ani13081308>

Kandel, P., Vanderick, S., Vanrobays, M. L., Soyeurt, H., & Gengler, N. (2018). Consequences of genetic selection for environmental impact traits on economically important traits in dairy cows. Animal Production Science, 58(10), 1779–1787. <https://doi.org/10.1071/AN16592>

Lakamp, A. D., Ahlberg, C. M., Allwardt, K., Broocks, A., Bruno, K., McPhillips, L., Taylor, A., Krehbiel, C. R., Calvo-Lorenzo, M. S., Richards, C., Place, S. E., Desilva, U., Kuehn, L. A., Weaber, R. L., Bormann, J. M., & Rolf, M. M. (2023). Variance component estimation and genome-wide association of predicted methane production in crossbred beef steers. Journal of Animal Science, 101, skad179. <https://doi.org/10.1093/jas/skad179>

Lassen, J., & Løvendahl, P. (2016). Heritability estimates for enteric methane emissions from Holstein cattle measured using noninvasive methods. Journal of Dairy Science, 99(3), 1959–1967. <https://doi.org/10.3168/jds.2015-10012>

Layton, J., Finocchiaro, R., Benzoni, L., Van Kaam, J.-T., Tiezzi, F., Marusi, M., & Cassandro, M. (2025). Genetic aspects of methane emissions and feed intake in growing Holstein bulls. Italian Journal of Animal Science, 24(1), 1909–1914. <https://doi.org/10.1080/1828051X.2025.2550513>

Lopes, L. S. F., Schenkel, F. S., Houlahan, K., Rochus, C. M., Oliveira, G. A., Oliveira, H. R., Miglior, F., Alcantara, L. M., Tulpan, D., & Baes, C. F. (2024). Estimates of genetic parameters for rumination time, feed efficiency, and methane production traits in first-lactation Holstein cows. Journal of Dairy Science, 107(7), 4704–4713. <https://doi.org/10.3168/jds.2023-23751>

Manzanilla-Pech, C. I. V., Stephansen, R. B., Difford, G. F., Løvendahl, P., & Lassen, J. (2022). Selecting for feed-efficient cows will help to reduce methane gas emissions. Frontiers in Genetics, 13, 885932. <https://doi.org/10.3389/fgene.2022.885932>

Manzanilla-Pech, C. I. V., De Haas, Y., Hayes, B. J., Veerkamp, R. F., Khansefid, M., Donoghue, K. A., Arthur, P. F., & Pryce, J. E. (2016). Genomewide association study of methane emissions in Angus beef cattle with validation in dairy cattle1. Journal of Animal Science, 94(10), 4151–4166. <https://doi.org/10.2527/jas.2016-0431>

Mc Court, A., Yan, T., Mayne, C. S., & Porter, M. G. (2006). Prediction of methane output in beef cattle from indirect respiration calorimetry data. International Congress Series, 1293, 46–49. <https://doi.org/10.1016/j.ics.2006.01.073>

Pickering, N. K., Chagunda, M. G. G., Banos, G., Mrode, R., McEwan, J. C., & Wall, E. (2015a). Genetic parameters for predicted methane production and laser methane detector measurements1. Journal of Animal Science, 93(1), 11–20. <https://doi.org/10.2527/jas.2014-8302>.

Pszczola, M., Calus, M. P. L., & Strabel, T. (2019). Short communication: Genetic correlations between methane and milk production, conformation, and functional traits. Journal of Dairy Science, 102(6), 5342–5346. <https://doi.org/10.3168/jds.2018-16066>

Renand, G., Vinet, A., Decruyenaere, V., Maupetit, D., & Dozias, D. (2019). Methane and Carbon Dioxide Emission of Beef Heifers in Relation with Growth and Feed Efficiency. Animals, 9(12), 1136. <https://doi.org/10.3390/ani9121136>

Ryan, C. V., Pabiou, T., Purfield, D. C., Kelly, D. N., Murphy, C. P., & Evans, R. D. (2025). Genetic correlations between enteric methane and traits of economic importance in a beef finishing system. Journal of Animal Science, 103, skaf162. <https://doi.org/10.1093/jas/skaf162>

Ryan, C. V., Pabiou, T., Purfield, D. C., Berry, D. P., Conroy, S., Murphy, C. P., & Evans, R. D. (2024). Exploring definitions of daily enteric methane emission phenotypes for genetic evaluations using a population of indoor-fed multi-breed growing cattle with feed intake data. Journal of Animal Science, 102, skae034. <https://doi.org/10.1093/jas/skae034>

Ryan, C. V., Pabiou, T., Purfield, D. C., Conroy, S., Kirwan, S. F., Crowley, J. J., Murphy, C. P., & Evans, R. D. (2022). Phenotypic relationship and repeatability of methane emissions and performance traits in beef cattle using a GreenFeed system. Journal of Animal Science, 100(12), skac349. <https://doi.org/10.1093/jas/skac349>

Silva, J. A., Valente, J. P. S., Mota, L. F. M., Rodrigues, G. R. D., Soares, T. L. S., Marcatto, J. O. S., Pelaez, A. M., Monteiro, F. M., Canesin, R. C., Albuquerque, L. G., & Mercadante, M. E. Z. (2025). Genetic Parameters of Methane Emission, Feed Efficiency, Feeding Behaviour, and Growth Traits in Beef Cattle. Journal of Animal Breeding and Genetics, jbg.70037. <https://doi.org/10.1111/jbg.70037>

Velazco, J. I., Herd, R. M., Cottle, D. J., & Hegarty, R. S. (2017). Daily methane emissions and emission intensity of grazing beef cattle genetically divergent for residual feed intake. Animal Production Science, 57(4), 627–635. <https://doi.org/10.1071/AN15111>
